# Characterisation of plasmodial transketolases and identification of potential inhibitors: an in silico study

**DOI:** 10.1186/s12936-020-03512-1

**Published:** 2020-11-30

**Authors:** Rita Afriyie Boateng, Özlem Tastan Bishop, Thommas Mutemi Musyoka

**Affiliations:** Research Unit in Bioinformatics (RUBi), Department of Biochemistry and Microbiology, Rhodes University, P.O. Box 94, Grahamstown, 6140 South Africa

**Keywords:** Transketolase, Malaria, *Plasmodium*, Molecular docking, Molecular dynamics simulation, Protein sequence analysis, Protein structural analysis

## Abstract

**Background:**

Plasmodial transketolase (PTKT) enzyme is one of the novel pharmacological targets being explored as potential anti-malarial drug target due to its functional role and low sequence identity to the human enzyme. Despite this, features contributing to such have not been exploited for anti-malarial drug design. Additionally, there are no anti-malarial drugs targeting PTKTs whereas the broad activity of these inhibitors against PTKTs from other *Plasmodium* spp. is yet to be reported. This study characterises different PTKTs [*Plasmodium falciparum *(*PfTKT*),* Plasmodium vivax *(*PvTKT*),* Plasmodium ovale *(*PoTKT*),* Plasmodium malariae *(*PmTKT*) and *Plasmodium knowlesi *(*PkTKT*) and the human homolog (*HsTKT*)] to identify key sequence and structural based differences as well as the identification of selective potential inhibitors against PTKTs.

**Methods:**

A sequence-based study was carried out using multiple sequence alignment, phylogenetic tree calculations and motif discovery analysis. Additionally, TKT models of *Pf*TKT, *Pm*TKT, *Po*TKT, *Pm*TKT and *Pk*TKT were modelled using the *Saccharomyces cerevisiae* TKT structure as template. Based on the modelled structures, molecular docking using 623 South African natural compounds was done. The stability, conformational changes and detailed interactions of selected compounds were accessed viz all-atom molecular dynamics (MD) simulations and binding free energy (BFE) calculations.

**Results:**

Sequence alignment, evolutionary and motif analyses revealed key differences between plasmodial and the human TKTs. High quality homodimeric three-dimensional PTKTs structures were constructed. Molecular docking results identified three compounds (SANC00107, SANC00411 and SANC00620) which selectively bind in the active site of all PTKTs with the lowest (better) binding affinity ≤ − 8.5 kcal/mol. MD simulations of ligand-bound systems showed stable fluctuations upon ligand binding. In all systems, ligands bind stably throughout the simulation and form crucial interactions with key active site residues. Simulations of selected compounds in complex with human TKT showed that ligands exited their binding sites at different time steps. BFE of protein–ligand complexes showed key residues involved in binding.

**Conclusions:**

This study highlights significant differences between plasmodial and human TKTs and may provide valuable information for the development of novel anti-malarial inhibitors. Identified compounds may provide a starting point in the rational design of PTKT inhibitors and analogues based on these scaffolds.

## Background

Malaria remains a major public health problem in the tropical regions of Africa, Eastern Mediterranean, West Pacific, South America and South-East Asia, resulting in nearly half a million fatalities annually [1]. Despite the numerous anti-malarial drugs developed so far, the recurrent ability of the *Plasmodium* parasites to develop resistance against all existing chemotherapies remains the greatest challenge towards global malaria eradication [1]. This has prompted an urgent need for the discovery of alternative anti-malarial chemotherapies exhibiting novel modes of action.

**Fig. 1:**
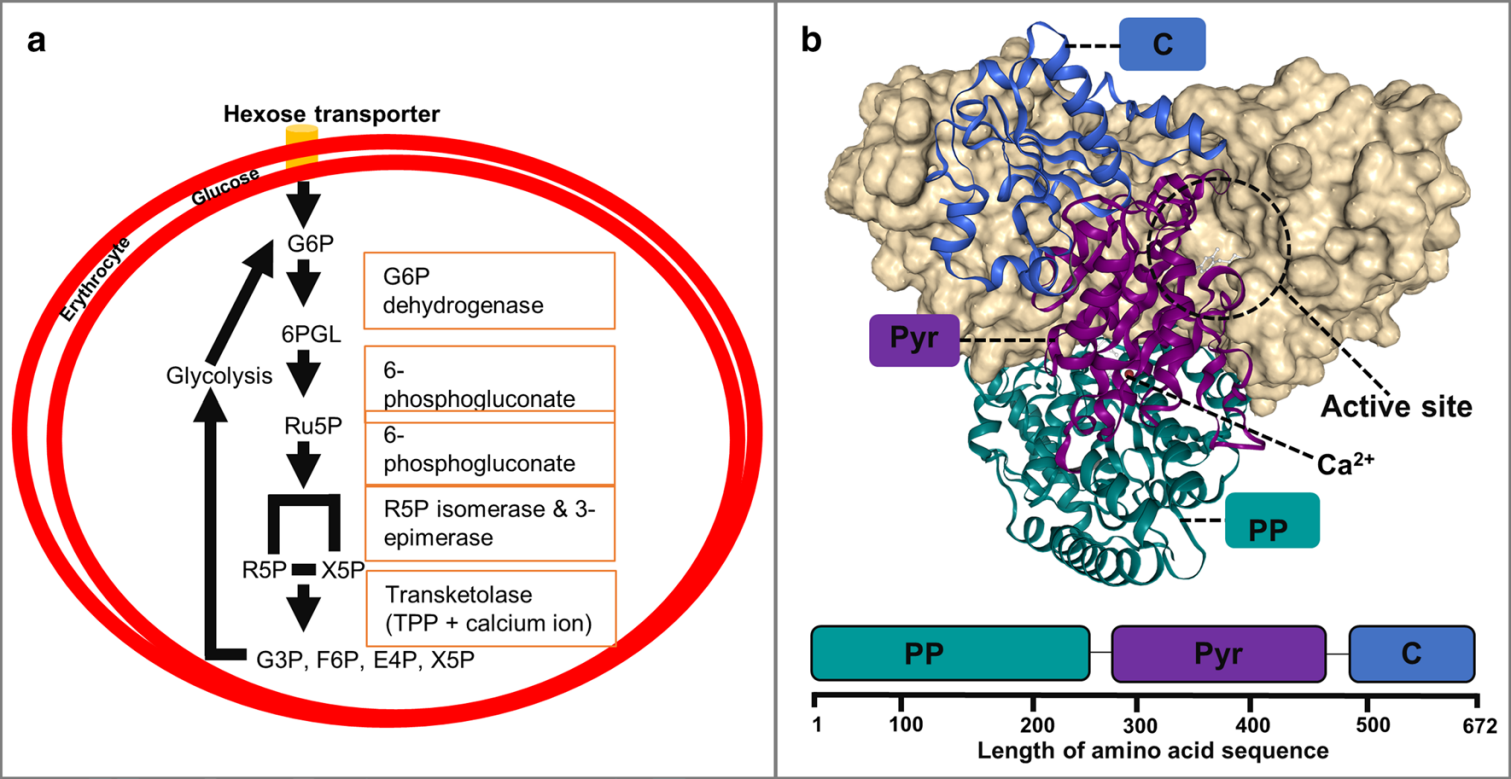
The role of TKT and domain arrangement in *Plasmodium* parasites. **a** Mechanism of TKT in *Plasmodium* parasites. **b** Domain arrangements (PP—pyrophosphate; Pyr—pyrimidine and carboxy—C-terminal) of TKTs using the *P. falciparum* TKT as the reference structure

Transketolase (TKT), a key enzyme essential for parasite survival, is one of the novel pharmacological targets being explored as potential anti-malarial drug target [2]. TKT is a vital enzyme in the non-oxidative phase of the pentose phosphate pathway (PPP) which primarily generates NADPH, pentoses and ribose-5-phosphate (R5P), vital in nucleotide and nucleic acid synthesis. The enzyme channels 2-carbon ketol units (CH_2_OH–CO–) from xylulose-5-phosphate to R5P or erythrose-4-phosphate (E4P) generating glyceraldehyde-3-phosphate (G3P), sedoheptulose-7-phosphate and fructose-6-phosphate (F6P) (Fig. 1a) [3]. TKT is expressed in a wide range of organisms, including humans [4] and *Plasmodium* parasites [2]. For its biological activity, it requires two co-factors, namely, thiamine pyrophosphate (TPP/ThDP) and divalent metal ions such as Ca^2+^ ion [5]. Similar to TKT of other species, plasmodial TKT is a homodimeric enzyme consisting of two identical monomers folded into a *V*-conformation structure upon cofactor binding [6, 7]. Each monomer comprises of three separate α/β domains: N or pyrophosphate (PP), middle or pyrimidine (Pyr) and C-terminal (Fig. 1b). The PP domain (residue 3–322) comprises parallel β-sheets involved in the recognition of cofactors, substrate, and contains the TPP motif. The Pyr domain (residue 323–538), which serves as the substrate-binding site, shares a common fold with the central β-sheets of the PP domain and comprises the TKT motif [8]. A unique characteristic of TKT protein family is the presence of two symmetric functional sites located at the Pyr domain of one monomer and the PP of the other monomer and vice versa [9]. The active site residues form a deep funnel-like cleft towards the exposed reactive C-2 group of ThDP [9, 10]. The C-terminal domain is made of 150 residues forming 5 stranded β-sheets. This region is distant from the functional sites and is believed to be involved in the regulation of enzymatic activity as well as the stereochemical control of substrate binding [8]. To date, no TKT crystal structure from any of the *Plasmodium* species (spp.) has been resolved. However, crystallized structures from *Homo sapiens* (*Hs*TKT), *Saccharomyces cerevisiae* (*Sc*TKT) and other organisms [11] already exist.

In humans, the enzyme primarily links PPP to the glycolysis pathway, channeling excess sugar phosphates and NADPH to organs involved in biosynthesis [4]. Despite these functions, *Plasmodium* parasites have developed additionally their own way of producing more R5P required for the synthesis of nucleic acid and generation of energy in the form NADPH, employing TKT in the reverse directional PPP mechanism by utilizing F6P and G3P [12]. A study conducted by Atemna and Ginsburg reported that infected erythrocytes showed an 82% increase in PPP activity compared to the host’s cell [13]. This was due to oxidative stress caused by the parasites that interfered with the host’s cells thus, presenting plasmodial transketolases (PTKTs) as target of interest for anti-malarial drug development. Furthermore, *Hs*TKT shares a low sequence identity with PTKTs of which features contributing to the similarity has not been exploited.

To date, few inhibitors have been shown to inhibit *P. falciparum* TKT (*Pf*TKT). However, the broad activity of these inhibitors against TKTs of different *Plasmodium* spp. is yet to be reported. Structural analogs of TPP (oxythiamine pyrophosphate [12] or thiamine thaizolone diphosphate [14]) studied as cofactor inhibitors of *Pf*TKT not only had poor activity but also poor selectivity against host cell [15]. In terms of substrate processing inhibition, an analogue of TKT [*p-hydroxyphenylpyruvate* (HPP)] has been reported as a reversible and competitive TKT substrate inhibitor [16]. Additionally, in-vitro and in-silico studies of several synthesized substrate inhibitors derived from hybrid *4-anilinoquinoline triazines* exhibited higher inhibitory potency than that of HPP [17]. However, none of these inhibitors has been approved as a candidate anti-malarial drug.

The current study utilizes bioinformatics approaches to determine major sequence and structural differences between PTKTs and *Hs*TKT with the ultimate aim of using this information to design selective inhibitors. Similar in-silico approaches have been used to design potent inhibitors targeting other anti-malarial enzymes with promising outcomes [18–20]. Here, multiple sequence alignment (MSA) analysis, phylogenetic tree calculations and motif analysis were performed to identify the conservation of residues, evolutionary relationship and recurring motifs presumed to have biological functions, respectively. Using homology modelling, high-quality homodimeric structures of human infecting (HI) PTKTs were generated. Identified motifs were mapped to structures including the *Hs*TKT to explore the overall differences. The results showed prominent differences between PTKTs and *Hs*TKT proteins at the residue and evolutionary level. Motif 6, 8, 12 and 16 were located at the substrate-binding pocket of PTKTs but not in *Hs*TKT. Differences in substrate binding sites could be a starting point for good selectivity in the development of drugs. These results formed the basis for the second part of the study in which potential substrate scaffolds of HI PTKT from the South African Natural Compound database (SANCDB) [21] were identified. Molecular docking experiments identified SANC00107, SANC00411 and SANC00620 compounds as hits that preferentially bound to the active site of all HI PTKTs with strong binding affinity but not in the active site of the *Hs*TKT. These protein–ligand complexes were subjected to 100 ns all-atom molecular dynamics (MD) simulations combined with binding free energy calculations to analyse protein–ligand stability, their conformational dynamics in a solvated environment and identify energy contributions of key residues. It was observed that the binding of all hit compounds to PTKTs reduced residue fluctuations especially for the residues surrounding the active sites.

In summary, this study reports unique features of PTKTs which can be exploited to design PTKT inhibitors with great selectivity against the human homolog. Additionally, the identification of novel and chemically diverse scaffolds provides initial hint for candidate PTKT inhibitors.

## Methods

**Fig. 2:**
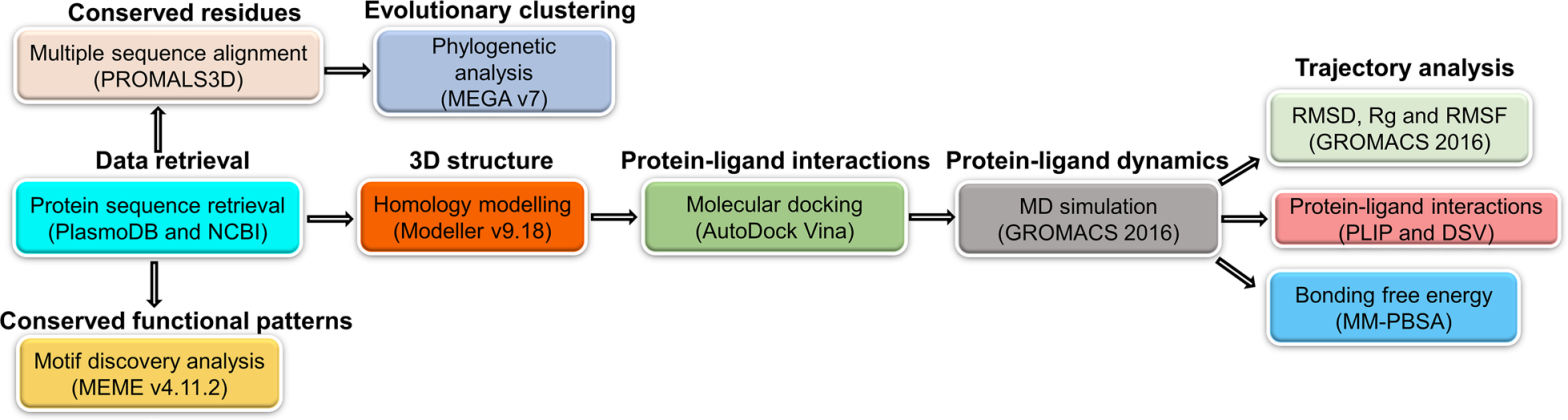
Graphical representation of the workflow of approaches and tools used in this study

The overall methodology and tools used in this study are graphically represented in Fig. 2.

### Transketolase sequence retrieval and multiple sequence alignment analysis

*Pf*TKT protein sequence (Accession ID: PF3D7_0610800) was retrieved from the *Plasmodium* Genomics Resources database (PlasmoDB version 33) [22]. Using *Pf*TKT as a query sequence, a set of homolog sequences from other *Plasmodium* species as well as from human were downloaded (Additional file 1). Additional TKT sequences from *Leishmania*, *Trypanosoma*, bacteria, fungi and *Anopheles* (*Plasmodium* parasite vector) were also retrieved from National Center for Biotechnology Information (NCBI) database [23] (Additional file 1). A reverse BLAST was performed to confirm that the retrieved sequences were indeed the true homologs (Additional file 2). All database searches were performed using the default parameter settings. Sequences with significant expect value (E) of zero or close to zero, coverage and significant sequence identity to *Pf*TKT were selected for subsequent analysis.

To determine the similarity and conservation of residues across the identified set of TKT proteins, multiple sequence analysis (MSA) was performed using PROfile Multiple Alignment with predicted Local Structures and 3D constraints (PROMALS3D) [24] and Multiple Alignment using Fast Fourier Transform (MAFFT) version 7 [25] web servers using default parameters. With PROMALS3D alignment, structural information from the 3D structure (PDB ID: 1TRK) of *Sc*TKT [11] was included. To determine the alignment accuracy, a comparison was made between the two outputs. Jalview version 2.0 [26] was used to visualize alignment outputs. Additionally, all versus all sequence identity of aligned sequences were calculated using an in-house Python script [27] and MATLAB software.

### Phylogenetic analysis of TKT sequences

To explore the evolutionary relationships among the identified TKTs, a phylogenetic tree was calculated using the PROMALS3D alignment as input and the Maximum Likelihood (ML) [28] method in the Molecular Evolutionary Genetics Analysis (MEGA) version 7 software package [29]. Bayesian information criterion (BIC) score was employed to estimate a reliable evolutionary model for tree calculation at complete (100%) and partial (95%, 90%) gap deletions. The top three evolutionary models with the lowest BIC scores for each gap treatment were then used in tree calculations. A thousand bootstrap replications and a very strong branch swap filter were applied during each tree calculation. A comparison between each generated tree and the corresponding bootstrap tree was performed to determine the best tree.

### 3D calculation of the plasmodial protein homodimers

Due to the unavailability of 3D structural data for the plasmodial TKTs, homodimeric structures of *P. falciparum *(*Pf*),* P. vivax *(*Pv*),* P. ovale *(*Po*),* P. malariae *(*Pm*) and *P. knowlesi* (*Pk*) TKTs in complex with TPP and Ca^2+^ cofactors were generated by homology modelling using MODELLER (version 9.18) [30] program. Prior to modelling, a suitable template based on the highest sequence identity and query coverage to target sequences was identified using PRotein Interactive MOdeling (PRIMO) protein structure prediction server [31]. Additionally, PROCHECK [32], Verify3D [33], QMEAN6 [34] and ProSA [35] tools were utilized to further validate the template. Based on these criteria, a homodimeric structure of *Sc*TKT in complex with cofactors and resolved at 2.0 Å (PDB ID: 1TRK) [11] was selected and retrieved from Protein Data Bank (PDB) [36]. Other TKT structures were additionally retrieved (Additional file 3). These were included in the modelling alignment stage to improve the alignment accuracy. Templates-target alignment from PROMALS3D was used to create “*pir*” files for modelling and included the two cofactors. For each target, 100 models were generated using a very slow refinement level. The resulting models were ranked using the Normalized Discrete Optimized Potential Energy (z-DOPE) scoring profile [37] and the top three models per protein validated as was with the template. Finally, the best model of each protein was selected based on a consensus result of the different validation tools. The crystal structure of *Hs*TKT in complex with TPP and Ca^2+^, resolved at 2.05 Å, PDB code 3OOY was retrieved for comparative analysis.

### Motif prediction and structural mapping

To elucidate functional and structural key patterns of the TKTs, motif analysis was performed via Multiple EM for Motif Elicitation (MEME) version 4.11.2 [38]. For the eighteen protein sequences dataset, unique motifs were searched with a maximum width of 6–30 residues. MEME output was parsed on the Motif Alignment and Search Tool (MAST) to check the significance of each identified motif as well as identify the overlapping motifs in the dataset. The MAST and MEME output files were then analysed using an in-house Python script [27] to generate a heatmap representing the occurrence and length of motifs at each position within the TKT family. The script utilizes Matplotlib, heatmap and Python algorithm to extract motif information from the MAST and MEME log data. Motifs identified were mapped onto their respective structures to assess the structural differences between PTKTs and *Hs*TKT.

### Molecular docking and drug-likeness analyses

In order to identify potential inhibitors against HI PTKTs, 3744 docking runs (6 proteins × 624 compounds) were performed on *Pf*TKT, *Pv*TKT, *Po*TKT, *Pm*TKT, *Pk*TKT and *Hs*TKT using AutoDock Vina [39]. This involved 623 minimized compounds from SANCDB [21] and HPP, the known TKT substrate inhibitor which was used as a positive control. Initially, docking validation was performed to assess the reproducibility of docking poses in AutoDock Vina. For the validation purpose, E4P substrate was re-docked to its co-crystallised structure (PDB code: 1NGS) and its binding pose compared to that of the initial complex. Prior to docking, small molecules and waters with an exception of the cofactors co-crystallised with *Hs*TKT (PDB: 3OOY) were removed using BIOVA Discovery studio [40]. The human enzyme (PDB code 3OOY) was selected based on its homodimeric state, few missing residues (3) and lower side-chain outliers (1.6%) compared to other existing structures in PDB. AutoDockTools 1.5.6 (ADT) [41] was used to prepare protein and ligand *pdbqt* input files where partial charges were assigned using the Gasteiger–Huckel method and all non-polar hydrogens merged. A + 2.0 charge was assigned to Ca^2+^ cofactor. A two-step docking protocol was then implemented. Firstly, blind docking runs using exhaustiveness of 320 and a cuboid box dimension of 120 × 120 × 120 with a grid spacing of 0.375 Å covering each protein was performed. TKT has two similar active sites and hence compounds that bound to these sites of the PTKTs and not to *Hs*TKT were extracted using BIOVA Discovery studio. Secondly, targeted docking was performed at the active site formed by the PP domain of one monomer (chain A) and the Pyr domain of the other monomer (chain B) using the compounds identified in the previous blind docking step. A grid box size of 30 × 30 × 30 covering the entire active site while grid box centered at x = − 6.17 Å, y = 55.14 Å and z = 19.83 Å with the default spacing and exhaustiveness of 192 was utilized. Compounds with binding energies lower than that of HPP were selected for further studies. Additionally, the Lipinski rule of five (RO5) [42] was performed to identify hits with drug-like features based on their molecular properties and structural features using the Supercomputing Facility for Bioinformatics and Computational Biology (SCFBio) [43] web server. To identify any occurrence of Pain Assay Interference Compounds (PAINS) structural features, selected compounds were screened using the PAIN-remover web server [44, 45]. The intermolecular interactions between the selected hits and each protein were determined using LigPlot+ [46] and Discovery studio [40] 2D plot.

### All-atom molecular dynamics simulations

MD simulations for 23 holo (protein with cofactors only) and holo–ligand bound complexes were performed in GROMACS (version 2016.4) package [47] to investigate system stability using an all-atom AMBER96 force field [48]. AMBER compatible topology files and parameters were generated using AnteChamber PYthon Parser interfacE (ACPYPE) [49]. MD simulations were carried out in two sets: the first and second sets included holo and holo–ligand bound complexes, respectively. For each complex, a dodecahedron box with a cutoff of 2.0 Å from the molecule edge was defined around the systems. Solvation was performed with the simple point charge (SPCE216) water model and the system’s net charge neutralized by adding 0.15 M NaCl. To obtain a correct structural geometry, energy minimization was performed by the steepest descent integrator at 0.01 energy step size as well as the process to reach a maximum force less than 1000 kJ mol^−1^ nm^−1^. All systems were equilibrated in a two-step canonical ensemble (each 100 ps); first under the NVT ensemble (number of particles, volume and temperature) fixed at 300 K using Berendsen temperature coupling and then NPT ensemble (number of particles, pressure and temperature) at 1 atm in all directions at a constant temperature of 300 K using the Parrinello–Rahman barostat algorithm [50]. Finally, all systems were subjected to 100 ns trajectory production runs with 0.002 ps timestep. Trajectories were analysed via various modules in GROMACS including *gmx rms*, *gmx rmsf* and *gmx gyrate* to determine the root mean square deviation (RMSD), root mean square fluctuation (RMSF) and radius of gyration (Rg) respectively. Additionally, system snapshots were generated at 20, 40, 60, 80 and 100 ns and the protein–ligand molecular interactions assessed using Protein–Ligand Interaction Profiler (PLIP) tool [51] and DS. Visualization of trajectories was done using the Visual Molecular Dynamics version 1.9.2 (VMD) tool [52] and system dynamic properties plotted using R-studio.

### Binding free energy calculations

The molecular mechanics Poisson–Boltzmann surface area (MM-PBSA) [53] method was utilized to predict the binding free energy (BFE) using the *g_mmpbsa* tool. MM-PBSA calculations are widely used in calculating BFE of protein–ligand interactions [54]. From each system trajectory, snapshots were obtained from the last 6 ns using a timestep of 50 ps. BFE was calculated as follows (Eqs. 1–4) [55].

1 $$\Delta G_{binding} = \Delta G_{complex} - \Delta G_{protein} + G_{ligand}$$

where $\Delta G_{complex}$ indicate the total BFE of protein–ligand and $\Delta G_{protein} + G_{ligand}$ represent isolated protein and ligand respectively in solution. However, BFE of each component is specified by:

2 $$\Delta G \le \Delta E_{Molecular mechanics } > - TS + < G_{Solvation} ,$$

3 $$\Delta {\text{E}}_{{{\text{molecular}}\;{\text{mechanics}}}} = \Delta {\text{E}}_{{{\text{covalent}} \left( {{\text{bond}} + {\text{angles}} + {\text{tortions}}} \right)}} + \Delta {\text{E}}_{{{\text{electrostatic}}}} + \Delta_{{{\text{vdW}} }} ,$$

4 $$\Delta G_{solvation} = \Delta G_{polar} + G_{non - polar} .$$

In polar solvation energy calculations, solute electric constant (pdie) was set to 4 due to the charged active site residues of TKT. Finally, the overall binding term was decomposed to identify key PTKT residues involved in the binding of ligands.

## Results and discussion

TKTs of seven *Plasmodium* species and the human homolog (*Hs*TKT) protein sequences were analysed. The plasmodial TKTs (PTKTs) were divided into two categories: TKTs of *P. falciparum*, *P. vivax*, *P. ovale*, *P. malariae* and *P. knowlesi* are referred in this study as human infecting (HI) PTKTs whereas *Plasmodium berghei* (*Pb*), *Plasmodium chabaudi* (*Pc*) and *Plasmodium yoelii* (*Py*) TKTs are referred as rodent infecting (RI) PTKTs. To increase the accuracy and robustness of the MSA and phylogenetic analysis, nine other non-*Plasmodium* sequences were also included. The general workflow of the study is divided into two parts. The first part focused on the identification of unique sequence and structural features between PTKTs and *Hs*TKT as determined by phylogenetic, MSA, motif search, homology modelling and motif mapping approaches. Based on the underlying differences, potential inhibitors against HI PTKTs were identified using molecular docking, MD simulations and BFE calculations.

### Part 1—characterization of TKT at the sequence and structural level

#### High residue conservation and clustering within plasmodial transketolases is observed

**Fig. 3:**
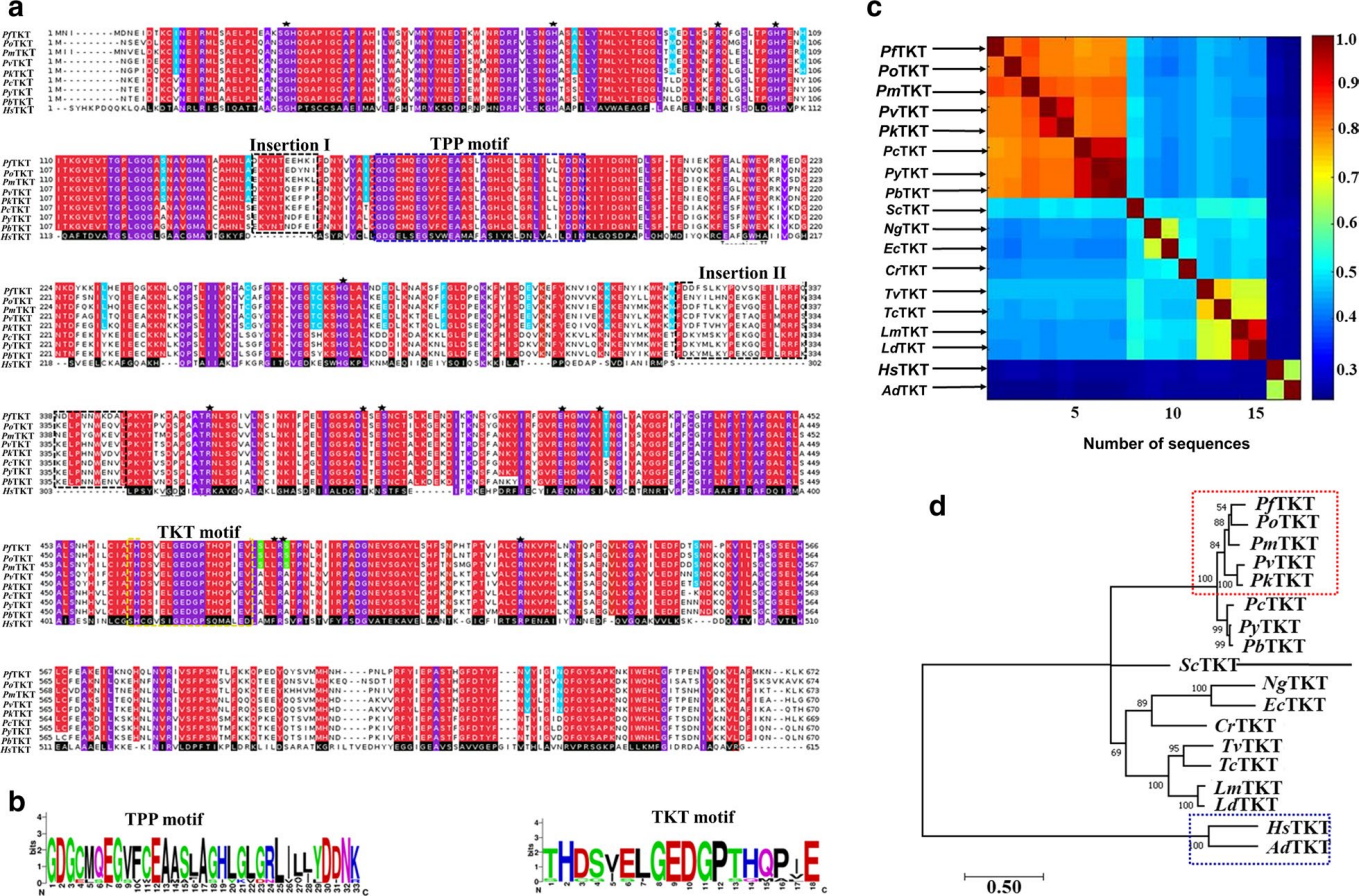
**a** PROMALS3D MSA results showing the conservation of all structural and functional residues. A violet color shows conserved residues in all aligned sequences whereas residues conserved in only PTKTs are indicated in red and their corresponding variations in *Hs*TKT in black. Distinct insertions in PTKTs are labelled as insertion I and II. All key residues are shown with an asterisk. **b** Detailed sequence logo representation of TPP and TKT motifs. **c** All versus all pairwise sequence identity calculations. **d** Phylogenetic tree derived using MSA from PROMALS3D. Maximum Likelihood method based on the Le_Gascuel_2008 model [28] was used for tree calculations at 90% gap deletions applying the Neighbor-Join and BioNJ algorithms. The tree with the highest log likelihood (− 11,888.05) is shown. Evolutionary relationships are shown in dashed boxes. Red; cluster of PTKTs. Cluster I and II indicate HI and RI *Plasmodium* sp. respectively. Blue; *Hs*TKT cluster with *Anopheles darlingi* TKT

Plasmodial and human TKTs were aligned using both PROMALS3D and MAFFT tools. MAFFT progressively aligns with scoring functions and other refining techniques while PROMALS3D uses profile to profile function that enables structural features to be used to guide the alignment process [24, 25]. Based on each alignment results profile, a more accurate alignment of loop regions, gaps and motifs was generated by PROMALS3D (Fig. 3a). MSA of TKT sequences revealed highly conserved residues, among all PTKT sequences compared to the human homolog. Previous MSA studies on TKTs have shown the presence of two highly conserved motifs (TKT and TPP), which are located in the PP and Pyr domains respectively. The TPP motif is characterised by ‘GDGxxxEGxxxExxxxAxxxxLxxLVxxxDxN’ signature [56]. In PTKTs, this motif starts with a charged residue sandwiched between two highly conserved hydrophobic residues ‘GDG’, followed by 21 less conserved protein residues (Fig. 3b). These residues have been shown to be vital in both the recognition and binding of the cofactor and substrate [3]. The TKT motif, a distinctive feature of members of this family consists of highly conserved ‘THDSIGLGEDGPTHQPIE’ residues and corresponded to protein sequences of position 463–480 (*P. falciparum* and *P. malariae*) and 460–483 (*P. ovale*, *P. vivax* and *P. malariae*). Among PTKTs, the TKT signature was highly conserved, while in *Hs*TKT, it was replaced by ‘SHCGVSIGEDGPSQMGLE’. Previous work by Schenk et al. [3] reported that the TKT motif is important for substrate binding and might contribute to the specificity of the binding of substrates between PTKTs and *Hs*TKT. The substrate-binding site of TKTs consists of acidic residues. In both PTKTs and *Hs*TKT, key acidic residues were highly conserved (shown in asterisks) except hydrophobic Leu residue (position 486—*P. falciparum numbering*), which is replaced with Phe residue in *Hs*TKT. Additionally, two unique inserts (insertion I and II) of about nine and thirty-one residues respectively occurred at the PP and Pyr domains of PTKTs and were absent in *Hs*TKT. Insertion II forms a long well-structured helix located at the active site and could contribute to its stability in PTKTs proteins. A previous study by Mitschke et al. [4] additionally reported similar insertions in *Sc*TKT but absent in *Hs*TKT.

On calculation of phylogenetic tree, the clustering based on eighteen TKT sequences agreed with all versus all sequence identity clustering as shown in Fig. 3c. A similar observation was previously shown in other families [57, 58]. Four clades viz I, II, III and IV were observed. Clade I and II consist of all *Plasmodium* species and share sequence identity above 76%. *Plasmodium* members separately branched out and grouped into two main clusters (I and II). In cluster I, TKT from *P. falciparum*, *P. ovale* and *P. malariae* grouped together. *P. falciparum* and *P. ovale* have been classified as mixed malaria infection species [59]. Additionally, *P. vivax* and *P. knowlesi* also formed a clade that indicates that they are highly conserved and evolutionary related. *P. vivax* and *P. knowlesi* have been reported to be closely related, which may indicate a possible host switch at some stage in evolution. In cluster II, *P. chabaudi*,* P. berghei* and *P. yoelii* TKTs clustered together. These species infect rodents and are valuable laboratory models for pathogen–host studies, vaccine and drug development [60]. The human and *Anopheles darlingi* TKTs by contrast grouped separately. A low sequence identity of 28% between the human TKT and PTKTs was observed. Earlier work by Mitschke et al. [4] showed a similar low sequence identity between *Hs*TKT and other TKTs. This could be attributed to the evolution of TKT, whereby the human homolog has been distantly related to the PTKTs through natural selection, random variations and genetic drift [61]. The results demonstrate evolutionary diversity between PTKTs and *Hs*TKT.

#### Accurate homodimer protein structures are built using homology modelling

Using PRIMO, the initial step of an appropriate template search was carried out. PRIMO integrates HHsearch [62] and protein BLAST [63] search tools with different alignment programs for precise predictions. The full-length *P. falciparum*, *P. vivax*, *P. ovale*, *P. malariae* and *P. knowlesi* TKTs in complex with TPP and Ca^2+^ were successfully modelled based on template *Sc*TKT, PDB ID: 1TRK. The template which was in complex with both TPP and Ca^2+^ had a resolution of 2.0 Å. The sequence identities between query sequence (targets) and template were greater than 47%, with 98% coverage (Additional file 3). For each protein, 100 models were generated, and initially assessed by the z-DOPE scores function [37]. The z-DOPE score is a normalized probability theory that implements statistical potential depending on atomic distance and relies on native protein structures [37]. A negative z-DOPE score is an indicative of near native structural models. The top three models with lowest negative z-DOPE scores were selected and model quality evaluation was done using ProSA, QMEAN6, Verify3D and PROCHECK web servers. ProSA tool performs global and local quality assessment of monomers and compares them with experimental data of native structures. At least each protein model was within the global z-score of below − 11.22 for each monomer. QMEAN6 compares models with the non-redundant set of 9766 high-resolution PDB chains and score as z-scores. From the results, each model quality evaluation z-score was at least 1.00, indicating models were near experimentally determined structures. The similarity of the three-dimensional (3D) profile atomic models with their respective amino acid residues predicted by Verify3D tool showed predicted scores more than 0.2 (above 80%) indicating an acceptable model. A Ramachandran plot showing the distribution of normal phi (ϕ), psi (ψ) and stereochemical properties of the entire structure of each protein was performed using on-line PROCHECK software. Each generated model had residues of more than 87.0% and 9.8% in the most favoured and allowed regions respectively indicating a good distribution of torsion angles (Table 1). In general, a consensus of these quality checks showed that the modelled structures were accurate and valid for structure-based analysis as outlined below.

**Table 1:** Quality evaluation scores of PTKT protein structures modeled

Protein	Modeller	ProSA		QMEAN6	Verify3D (%)	PROCHECK (%)		
	Z-DOPE score	z-Score monomers		QMEAN score	3D-1D score	Ramachandran (residues location)		
		A	B			Favoured	Allowed	Disallowed
*1TRK	− 2.01	− 12.93	− 12.87	0.94	92.00	89.20	10.80	0.00
*Pf*TKT	− 1.39	− 12.11	− 12.01	0.77	91.67	90.10	9.80	0.00
*Pv*TKT	− 1.40	− 12.33	− 12.25	0.77	91.34	88.30	10.90	0.10
*Po*TKT	− 1.35	− 11.28	− 11.27	0.76	84.30	88.40	10.90	0.20
*Pm*TKT	− 1.29	− 11.23	− 11.20	0.75	84.85	87.30	11.90	0.20
*Pk*TKT	− 1.35	− 11.58	− 11.48	0.77	85.10	89.20	9.80	0.30

*Indicates the evaluation scores of the template used in modeling

#### Unique motifs in plasmodial transketolases identified

**Fig. 4:**
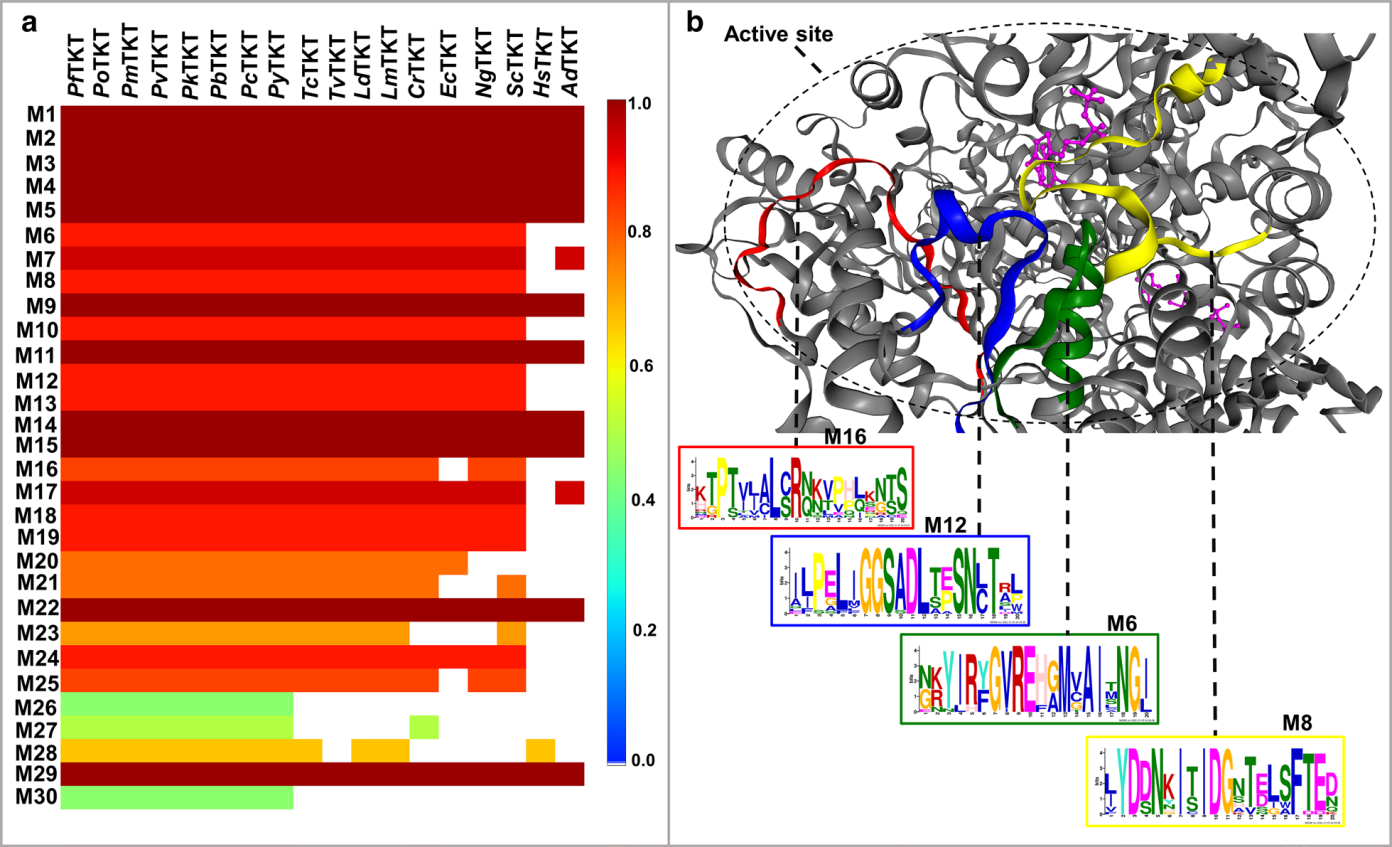
Motif discovery analysis of plasmodial and human TKTs. **a** A heatmap showing the conservation and distribution of identified different motifs. **b** A cartoon representation showing the location of motifs at the active site of PTKTs but absent in *Hs*TKT. Each motif information is shown in squared boxes. *Pf*TKT was used as the reference structure for mapping

Conserved motifs may play vital structural and functional roles [64]. This section aimed to identify unique motifs to PTKTs and absent in the human TKT. As there is no well establish rule about the length of the functional motifs, the short linear motifs criteria of 3–11 residues in length that are known to be functional ones in the protein–protein interfaces was applied [65]. The motif length predicted by MEME was adjusted to lengths 3–20 residues to incorporate potential motifs slightly longer than average. This range was also previously applied in identifying motif in enterovirus capsids [64]. Analysis of the dataset predicted a maximum of 30 non-redundant motifs. The conservation of each motif across the sequences is shown in Fig. 4a. Motif 1, 2, 3, 4, 5, 19, 11, 14, 15 and 21 were highly conserved across all TKT sequences, an indication of functional importance as previously reported [66, 67]. Despite the observed residue conservation in all TKTs studied, the TKTs of protozoan origin (including PTKTs) uniquely possed motif 6, 8, 10, 12, 13, 16, 18, 19, 20, 21, 24 and 25 which were conspicuously absent in *Hs*TKT. This explains the observed low sequence identity between the two protein groups. Motif 6, 8, 12 and 16 were located at the active site tunnel (Fig. 4b) and may be linked to substrate specificity. Interestingly, motif 6 and 8 (Pfam IDs: PF02779 and PF00456) respectively comprise the pyrimidine and TPP binding domain involved in recognition of cofactor and substrate [68]. Motif 10, 13, 19 and 20 were located at the C-terminal domain. Even though the functionality of the C-terminal domain remains unknown, it has been reported to be important in ensuring TKT’s stereochemistry towards the substrate [15]. Motif 26, 27 and 30 were only present in PTKTs, but absent in all other TKTs. Even though their functions are still unknown, motif 26 forms a well-structured α-helix which could be linked to structural stabilization. The specific details of the identified motifs and their location are in Additional file 4. In light of the observed unique motif composition in PTKTs, a possibility of achieving selective drugs targetting these plasmodial enzymes exist and a detailed discussion forms the second part of this study.

### Part 2—identification of potential hit compounds

#### Three SANCDB compounds identified against human infected PTKTs

**Fig. 5:**
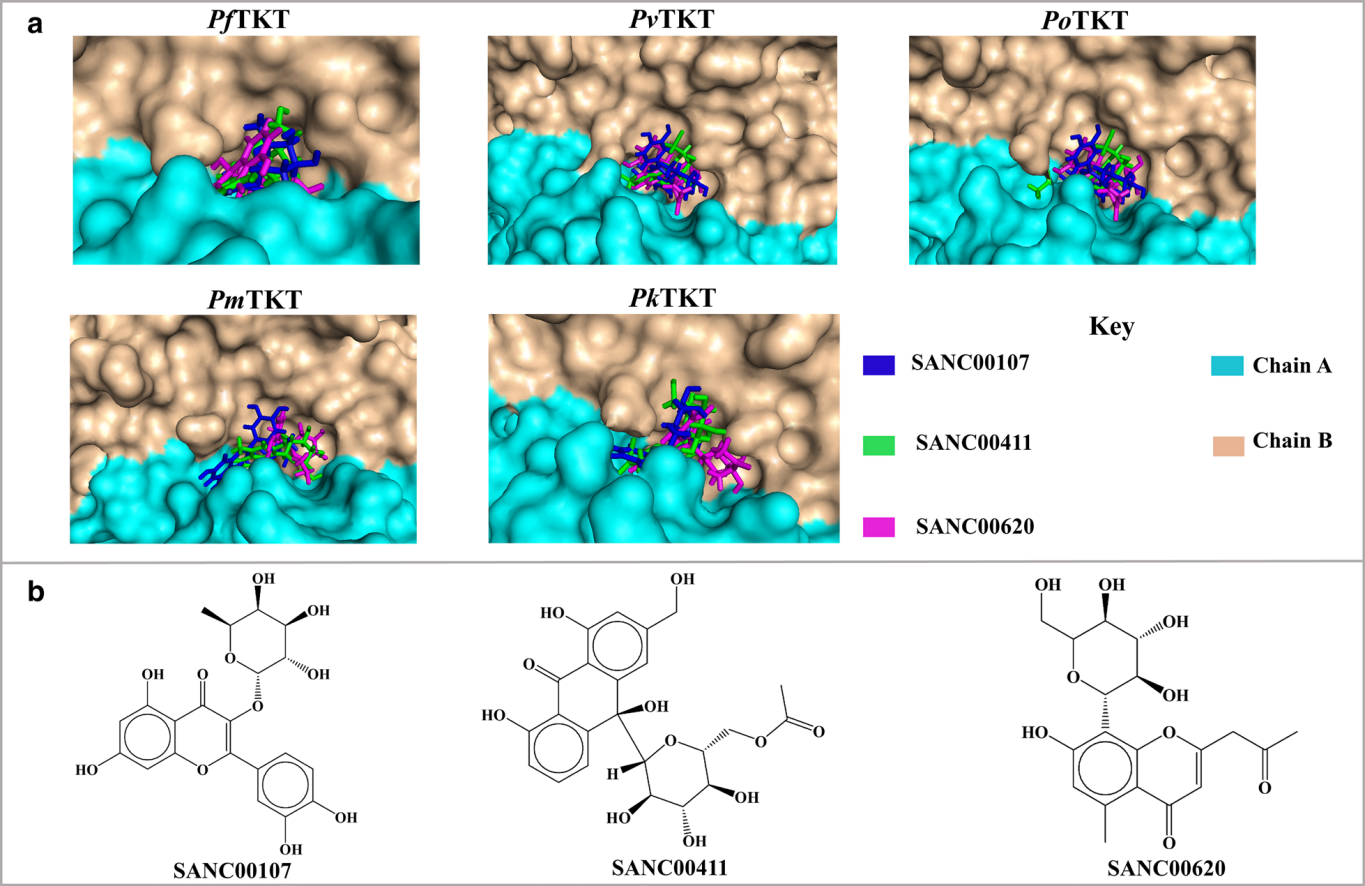
Binding poses of identified hit compounds in the active site of HI PTKTs. Binding orientation of SANC00107 (blue), SANC00411 (green) and SANC00620 (violet). **b** The 2D representation of compounds

Natural products and their unique scaffolds have been an important start point for drug discovery [69]. In this study, 623 South African natural compounds were examined using structure-based docking approach. Several studies have successfully used this kind of approach to identify hit compounds against various *Plasmodium* protein targets [70, 71]. During the screening process, HPP (a known TKT inhibitor) was included as a positive control to guide in the selection of potential hits. Molecular docking was carried out using AutoDock Vina which implements various stochastic algorithms to predict the binding orientation of ligands in a specific protein pocket. Additionally, AutoDock Vina has been tested against high throughput virtual screening of the Directory of Useful Decoys (DUD) [72] and noted to be a strong competitor against other screening programs in detecting binding poses [39]. Initial re-docking of the substrate erythrose-4-phosphate (E4P) to *S. cerevisiae* [in complexed with thiamine pyrophosphate (TPP) and Ca^2+^], PDB ID:1NGS generated a reproducible pose to the co-crystallised E4P when superimposed, authenticating the docking protocol. Analysis of docking results showed that 22 compounds bound in the active site of all PTKTs. The binding of these ligands against the human homolog was also predicted in order to ensure specificity towards PTKTs was achieved. Out of the 22 compounds, SANC00132, SANC00133, SANC00135, SANC00119, SANC00123, SANC00134 and SANC00121 bound in the active sites of *Hs*TKT. Thus, these compounds were left out in further analysis. Further screening of potential compounds was carried out on the basis of molecular interactions with conserved charged, polar and hydrophobic catalytic residues and the reactive C2 atom of TPP vital for catalysis of substrates. Overall, SANC00107 (*quercetrin-3-O-rhamnoside*), SANC00411 (*aloeresin*) and SANC00620 (*10-hydroxyaloin B 6′-O-acetate*) formed molecular interactions with TPP and known key residues also identified as part of the motif 6, 8, 12 and 16. Residue Ser29, Gly30, His31, Arg96, His266, Gly267, Arg361, Ser388, His465 and Arg524 (*Pf*TKT residue numbering) were highly conserved among the PTKTs (Fig. 3a), indictating a possible role in substrate binding. Additionally, residue Ser388 and Arg524 form part of unique motifs conserved in the active site of PTKTs but absent in *Hs*TKT (motif 12 and 16 respectively). Hydrogen bonds are important in a variety of biological processes, according to classical studies, so the sum of such bonds will play a fundamental role in determining the specificity of the molecule’s interaction with the pharmacological protein [73, 74]. Additional hydrophobic, van der Waals and pi–pi stacked interactions with catalytic residues and cofactor TPP observed could contribute to complex stability (Additional files 5, 6, 7). Interestingly, interactions between the functional amino acids of the aromatic ring Phe438, His93 and other non-aromatic residues Gly267, Arg96, Ile194 and Glu387 (*P. falciparum* residue positions) were also observed. These residues are highly conserved in PTKTs and form part of the functional motif 8 and 12 which are important for substrate binding as well PTKTs structure stability. From docking analysis, SANC00107, SANC00411 and SANC00620 displayed interesting results and may have a significant anti-malarial effect on HI PTKTs (Fig. 5 and Table 2). A significant principle in early drug development is decreasing drug failure rate by early identification and elimination of hits that consist of unfavorable structural and physicochemical properties that may result in reduced bioavailability and toxicity [75, 76]. Therefore, analysis to determine the drug-likeness of the identified hits was performed using the Lipinski RO5 and PAIN filtering. Lipinski RO5 provides a heuristic guide for predicting the oral bioavailability of a compound. According to the Lipinski RO5 filter, a drug-like compound must meet at least four of its standard requirements [42]. From the results, all the hits displayed drug-like properties (Table 3). Additionally, the PAIN filter showed that compounds lacked PAINS substructural features that could result in promiscuous compound activity on multiple protein targets [77].

**Table 2:** Binding energies of hit compounds in their respective binding proteins

	Hit compounds	TKT proteins								
		*Pf*	*Pv*	*Po*	*Pm*	*Pk*	*Pc*	*Pb*	*Py*	*Hs*
Binding affinity (Kcal/mol)	SANC00107	− 8.4	− 7.9	− 8.1	− 7.8	− 7.3	− 8.0	− 8.4	− 8.0	Not binding at active site (− 7.7)
	SANC00411	− 8.4	− 7.3	− 8.1	− 7.0	− 7.7	− 7.2	− 7.7	− 7.8	Not binding at active site (− 7.5)
	SANC00620	− 8.5	− 7.8	− 8.7	− 8.5	− 8.0	− 7.9	− 8.0	− 7.9	Not binding at active site (− 7.5)
	HPP	− 5.3	− 5.1	− 5.2	− 5.3	− 5.7	− 6.1	− 5.6	− 5.9	Binding at active site (− 5.9)

Control compound is indicated as HPP

**Table 3:** Druglikeness scores of hit compounds

Compounds	MW	HBD	HBA	LV	LogP	OB	PAINs filter
SANC00107	448.38	4.00	11.00	1.00	0.86	Good	Accepted
SANC00411	394.37	5.00	9.00	0.00	− 1.28	Good	Accepted
SANC00620	476.43	7.00	9.00	1.00	− 1.18	Good	Accepted

*MW* molecular weight, *LogP* octanol/water partition coefficient, *HBD* hydrogen bond donor, *HBA* hydrogen bond acceptor, *LV* Lipinski violation, *OB* oral bioavailability, *PAINS* pan-assay interference compounds

#### General information on potential hit compounds

Quercetrin (*quercetrin-3-O-rhamnoside*) compound (SANCDB ID: SANC00107) is a flavonoid from the leaf extract of *Combretum apiculatum* and is traditionally used in the treatment of anti-inflammation conditions [78] and antibacterial infections [79]. SANC00411 is an *Aloeresin* which originate from the Aloe family. The compound is classified as a coumaroylaloesin or a glycoside [80]. Its medical use is still unknown. However, *Aloeresin* is used in alcoholic beverages as flavoring agent. SANC00620 (*10-hydroxyaloin B 6′-O-acetate*) is an oxanthrone from *Aloe claviflora*. *Aloe claviflora* (*Aloaceae*) is the only species of Aloe that occurs in Strydenburg, South Africa, Free State Province [81]. Its use is still not experimentally known. However, the aloe family have recently emerged as to contain investigational anti-malarial compounds [81].

#### Protein–ligand stability determined using molecular dynamic studies

**Fig. 6:**
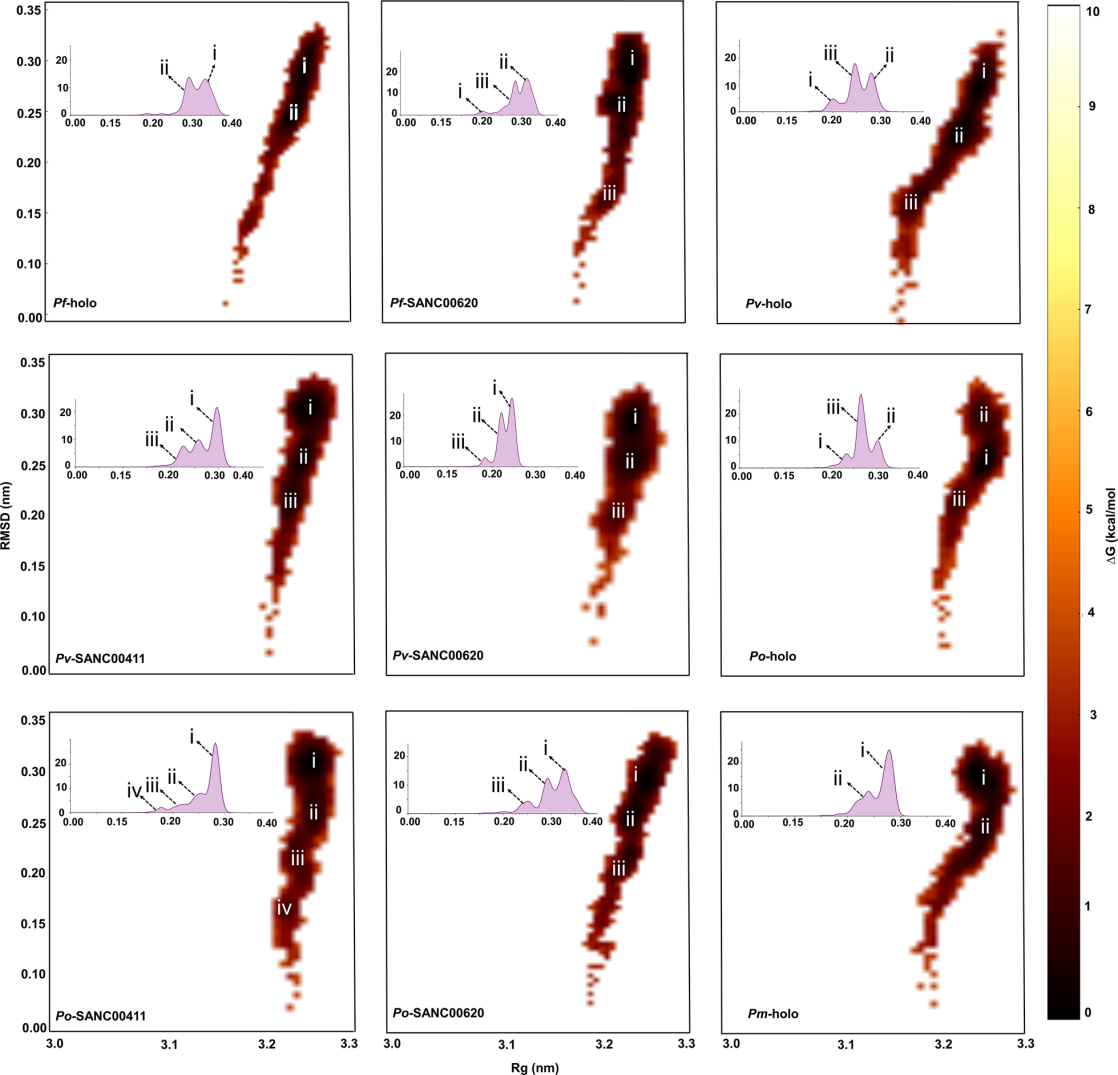
Free energy landscapes of major conformational distribution of each system snapshot determined using the Boltzmann constant and plotted along the RMSD and Rg values. In the systems showing more than one stable conformation (lowest energy), structure snapshots were generated in each energy minima and visualized using PyMOL

**Fig. 7:**
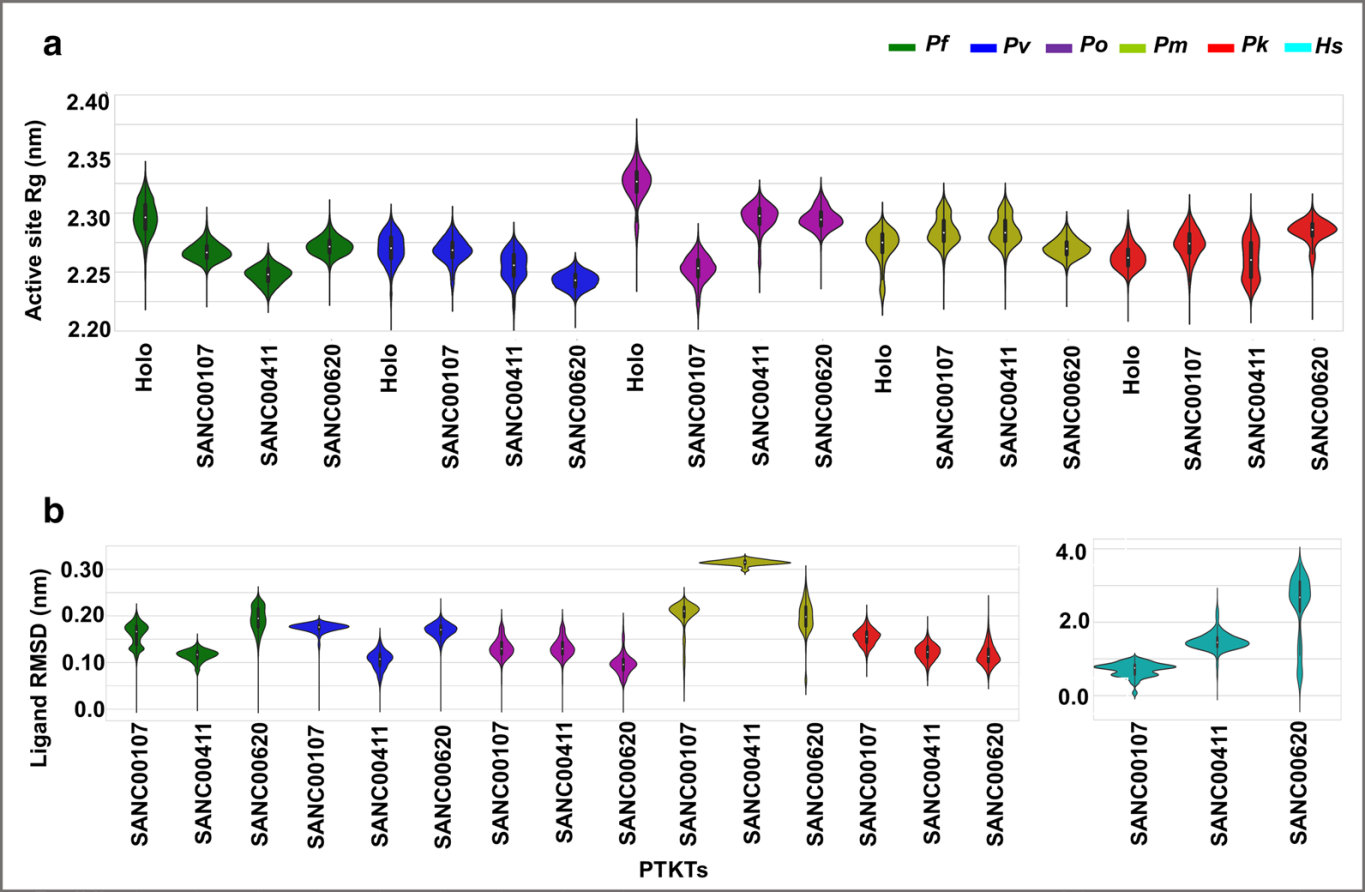
Kernel density distributions of **a** Rg of active site pocket and **b** ligand RMSD across both holo and holo-bound complexes

MD simulations remain one of the most powerful and reliable computational methods to evaluate the dynamic properties of biological systems in computer-aided drug design [82, 83]*.* Unlike in docking, MD simulations allow the systems to be subjected to physiological-like environment, allowing accurate description of the events happening during molecular recognition process. Herein, 100 ns MD simulations were performed on both the holo (protein with cofactors only) and holo–ligand bound complexes systems. From the different global and local dynamic analysis, a comparison (holo as reference) was performed to determine the conformational changes due to the binding of ligands. From the results (Fig. 6 and Additional files 8, 9, 10), all proteins with the exception of *P. falciparum* displayed similar Cα profiles between the holo and holo-bound complexes. In *P. falciparum*, the holo form displayed higher Cα RMSD profiles averaging at 0.32 nm in comparison to the holo–ligand bound systems. However, upon binding of the different ligands, decreased RMSD profiles similar to the other proteins were obtained (Additional file 11). TKTs are characterised by the presence of numerous loop regions connecting the various α-helices forming the central core of the proteins. Visualization of the different trajectories revealed a high conformational variability of the loop regions whereas the central core of proteins had vibration-like movements. To determine the conformational space sampled by each system during simulation, RMSD distribution plots were prepared using R. From the results, all the holo systems with the exception of *P. knowlesi* holo-protein displayed bi or multimodal distribution profiles. Upon the binding of the different ligands, a differential conformational distribution was noted between the holo and holo–ligand bound complexes (*P. falciparum* SANC00620, *P. vivax* SANC00411 and SANC00620, *P. ovale* SANC00411 and SANC00620, *P. malariae* SANC00411 and SANC00630 and *P. knowlesi* SANC00411). In the holo–ligand bound form, complexes with SANC00107 showed the least conformational variability except in *P. knowlesi*. To determine the cause of the observed conformational diversity, the free energy of each system snapshot was determined using the Boltzmann constant (Eq. 5) and plotted along the RMSD and Rg (a dynamic metric for determination of protein compactness) order parameters.

5 $$\Delta G\left( R \right) = - k_{B} T\left[ {InP \left( R \right) - InP_{\max } } \right].$$

where *K*_*B*_ is the Boltzmann constant and *P* is the probability distribution along order parameters (RMSD and Rg). In the systems showing multiple stable conformations (lowest energy), representative structure snapshots from each energy minima were generated and visualized using PyMOL. As observed in VMD, the notable conformational differences were due to the highly dynamic loop regions. Using all-atom Rg, the effect of ligand binding on the active site environment per system was calculated. As seen from the results (Fig. 7a), the binding of ligands stabilized the active site in all systems as denoted by the reduced Rg values when compared to the holo proteins. In *P. vivax*, *P. malariae* and *P. knowlesi* SANC00411 complexes, a bimodal distribution was observed which could be attributed to the flexibility of the active site loop region (*P. falciparum* numbering-residue 260–267). Additional analysis to calculate the conformational variability of each ligand pose over the simulation period was performed using its RMSD (Fig. 7b). From the results, majority of the compound systems had unimodal RMSD distributions an indication they remained stably bound onto the protein active pocket. However, a larger RMSD distribution (0.30 nm) was observed in *P. malariae* SANC00411 complex. Visualization of the trajectories in VMD to ascertain the cause of the observed bimodal ensembles revealed a flip-flop movement of the compound’s ester tail region. Finally, to determine if the selected ligands formed stable complexes with the *Hs*TKT, MD simulations were performed and the ligand RMSDs evaluated in a similar manner. From the results (Fig. 7b), all three compounds exited the binding pocket at different time points (20, 25 and 70 ns in the order of increasing compound ID). The current observation further strengthens the possibility of the identified compounds showing selectivity inhibition against the PTKTs.

#### Local residue flexibility as depicted by Cα RMSF

**Fig. 8:**
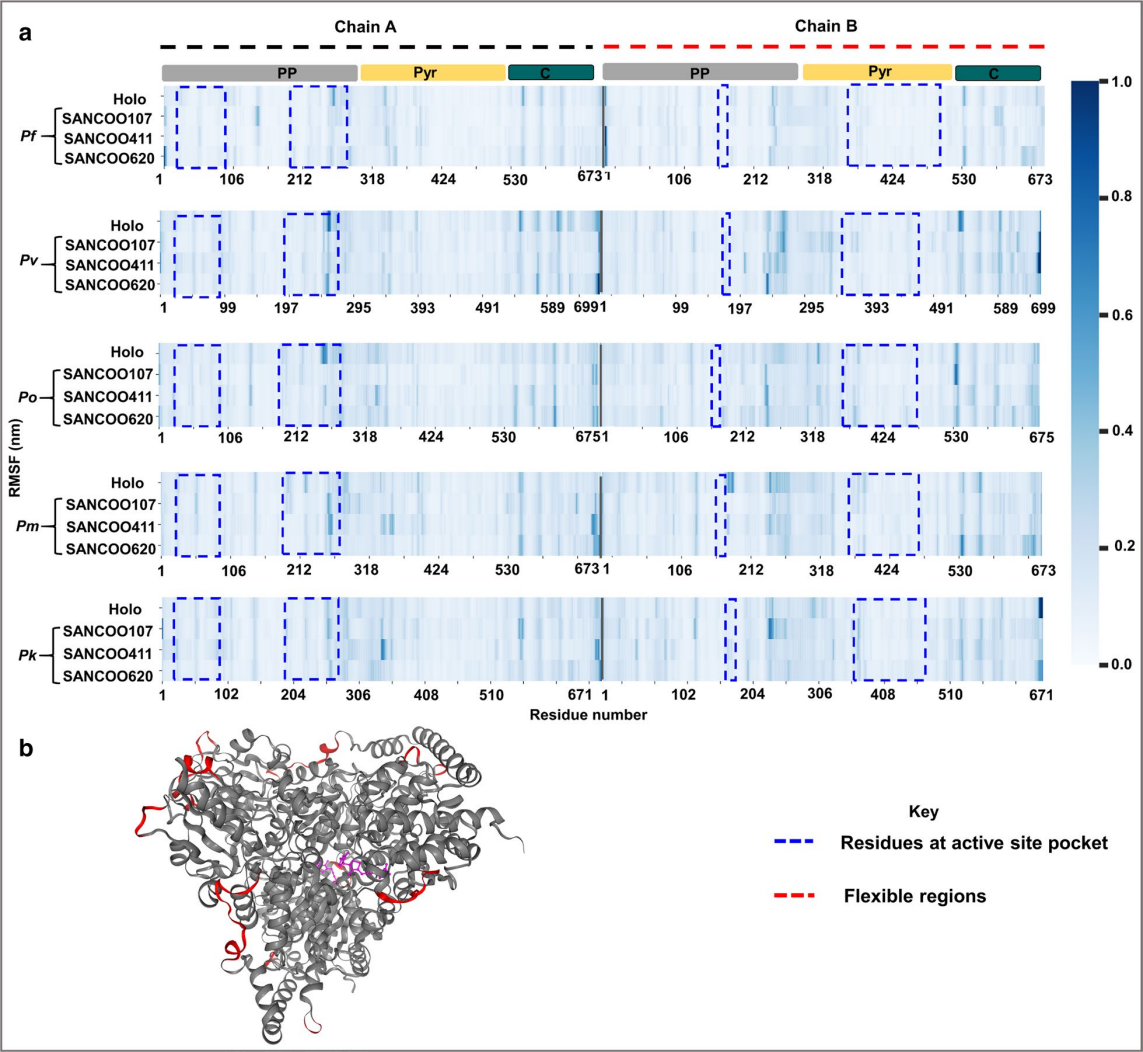
Per residue fluctuation in systems. **a** Heatmap of per residue RMSF of each monomer in both holo and holo–ligand bound complexes. The active site residues are shown in blue dashed lines. **b** Cartoon representation of the *Pf*TKT structure showing highly flexible regions

**Fig. 9:**
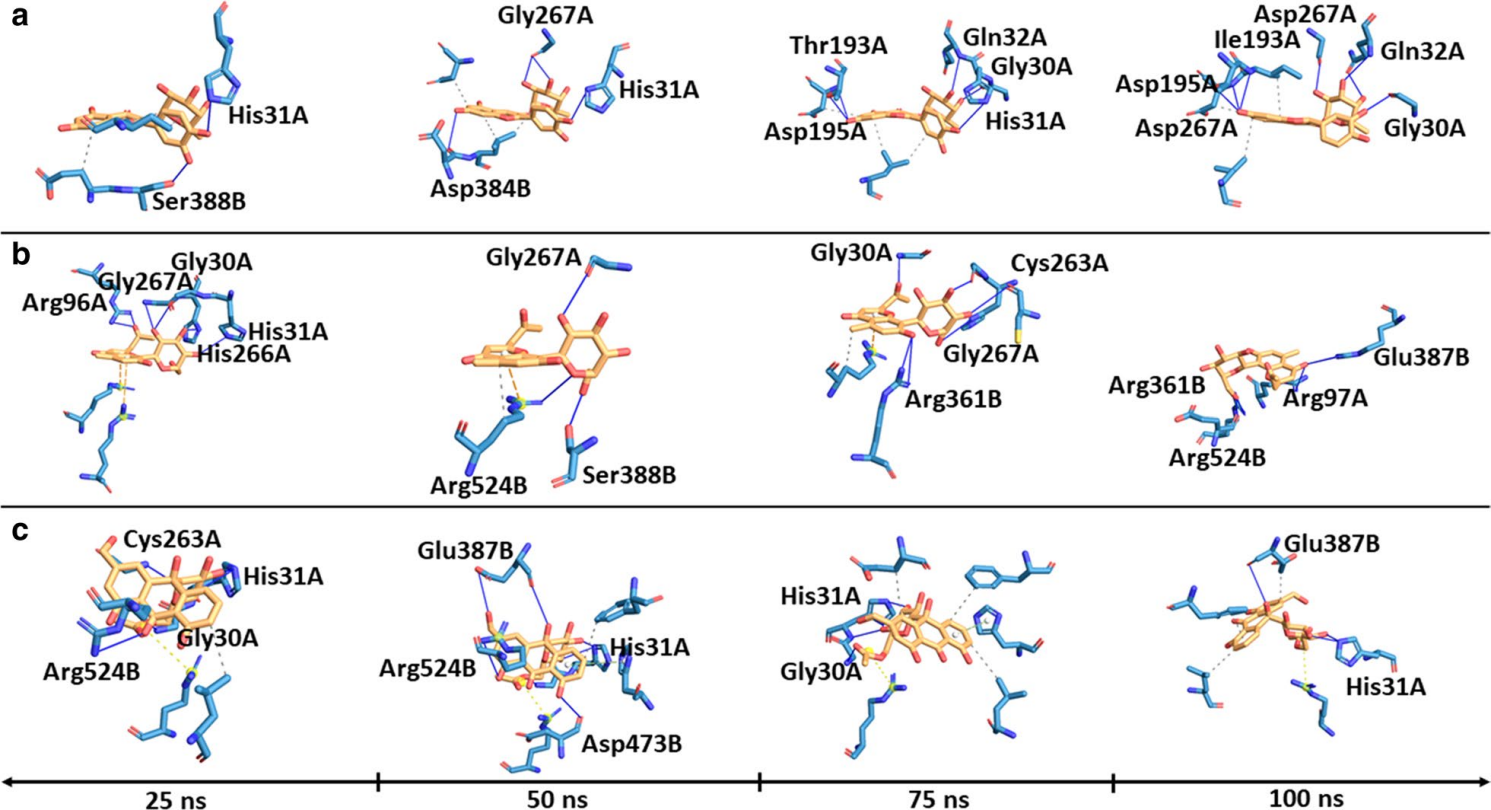
Time-dependent hydrogen bonds formed with *Pf*TKT. Hydrogen bonds are indicated as blue dashed lines were calculated at 25, 50, 75 and 100 ns snapshots using the PLIP tool. The protein–ligand interactions are: **a** *Pf*TKT-SANC00107; **b** *Pf*TKT-SANC00411; and **c** *Pf*TKT-SANC00620

Using RMSF, the per-residue flexibility was determined in each system and a comparison made with the corresponding holo structure (reference) (Fig. 8). Generally, RMSF for both holo and holo–ligand bound complexes exhibited high flexibility between residues 54–58, 144–147, 159, 194–196, 200–202, 254–264, 271–291, 301–306, 343–337, 354–357, 396, 549–554, 609, 660–668 (*P. falciparum* numbering). These residues form loops on the surface of the protein and as such the high mobility (Fig. 8b). Similar fluctuation patterns in both chain A and B were observed from the PP and C-terminal domains than the Pyr domain. In all three holo–ligand bound complexes, ligand binding decreased the flexibility of residues 254–264 and 549–554 (Fig. 8a). Residue 254–264 form a loop at the active site and may be critical for active site stability. In *P. malariae *SANC00107 and SANC00411 bound complexes, a higher flexibility was observed in residue 54–58, 145, 146, 159, 200, 202, 255–261, 271–291, 301–306, 355 and 356 396. These are loop regions located on the protein surface and hence confer such observed mobility [84] with an exception with residue 255–261. Residues identified at the active site showed decrease flexibility except for residues 255–261 (*P. falciparum* numbering) in *P. vivax* SANC00620, *P. malariae* SANC00107 and SANC00411. A similar observation was described by Yu et al*.* [85]. Since molecular interactions play a crucial role in the structural stability of the protein–ligand complexes, system snapshots (time step = 20, 40, 60 and 100 ns) were generated and molecular interactions between the ligand and protein determined. A number of hydrogen, hydrophobic and pi–pi stacking interactions with key residues were observed (Fig. 9 and Additional files 12, 13, 14, 15). These interactions were consistent with those identified in molecular docking studies (Additional files 7, 8, 9).

#### Binding free energy calculation favored holo–ligand complexes binding

**Fig. 10:**
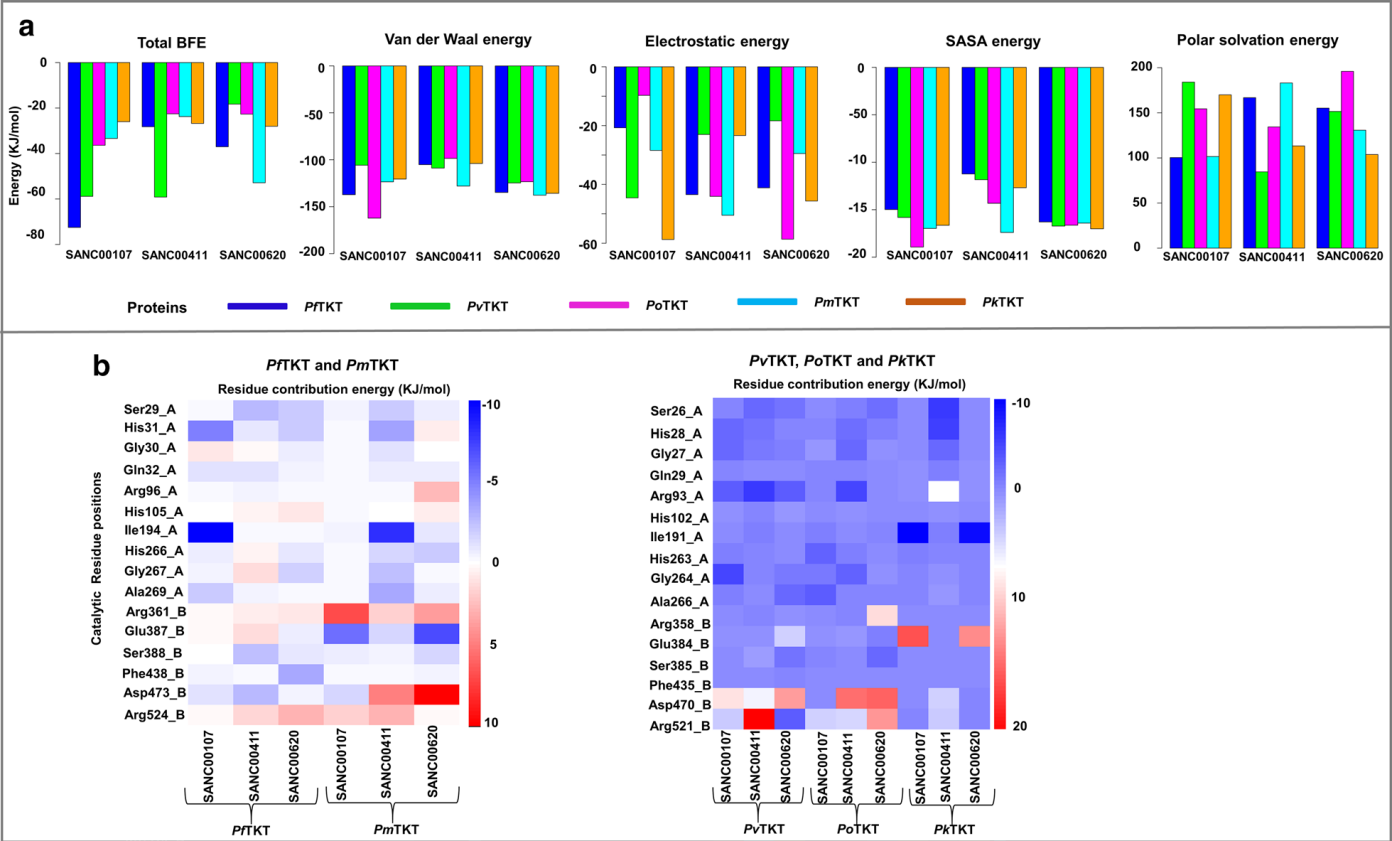
The energetic components of holo–ligand complex systems. **a** A summarized bar plots of binding free energies and individual energy components of hits compounds bound to *P. falciparum*, *P. vivax*, *P. ovale*, *P. malariae* and *P. knowlesi* TKTs derived by MM-PBSA. **b** The energetic contribution of key catalytic residues to BFE. Underscore A and B indicate residues from chain A and B respectively. Plots were generated from the R-studio program

To determine the strength of protein–ligand complexes, BFE was calculated using the MM-PBSA tool. Figure 10a shows a summary of the overall BFE and contribution of each energetic component. From the results, BFE for SANC00107, SANC00411 and SANC00620 ranged from − 73.00 to − 26.00 kJ/mol, − 60.00 to − 23.00 kJ/mol and − 53.00 to − 19.00 kJ/mol, respectively across systems. These predictions were in agreement with the binding energies obtained from AutoDock Vina. Additionally, the total van der Waals and electrostatic energies strongly favored holo–ligand bound complexes, suggesting that hydrophobic and electrostatic interactions respectively were important during the binding events. Energy contribution to non-polar solvation in all systems was slightly lower and comparable. The polar solvation component impaired the BFE, which could be attributed to the volume of the active site and compounds were therefore exposed to the solvent. Subsequent BFE decomposition was carried out to further determine the energetic contribution of each residue. From the results (Fig. 10b), residues from the PP and Pyr domains made a significant contribution to the binding. This pattern also confirms that PTKTs have a dimeric functional site, which is contrary to a study conducted by Hasan et al*.* [86] which predicted the 3D structure of *Pf*TKT as a monomer. Contribution per residue scores of key residues are displayed in Fig. 10b. In one monomer (Chain B), the Pyr domain residues had significantly varying energies (positive or negative) for the various compounds. In addition, the most positive energy contribution was shown by Arg524 in *P. falciparum* and *P. malariae* (residue number 521 in *P. vivax*, *P. ovale* and *P. knowlesi*). This can be attributed to Arg’s highly positive acid dissociation constant (pKa) which restricts the ionization of its side chains [87]. These data could be essential to understand the contribution of key residues to BFE during the discovery of PTKT inhibitors.

## Conclusion

Due to drug resistance and selectivity issues in malaria eradication efforts, it is imperative to characterise novel targets and discover new anti-malarials. This study aimed at identifying unique features between *Plasmodium* and human TKTs and additionally screen a natural compound database for PTKT inhibitors. Phylogenetics analysis showed distinct evolutionary distance between PTKTs and *Hs*TKT which overlapped with predicted pairwise sequence identities where all *Plasmodium* sp. showed above 77% sequence identity to *Hs*TKT (28%). Additionally, the alignment of TKT sequences highlighted well-conserved residues at the PP and Pyr terminal domains involved in substrate binding, whereas less conserved regions were seen at the C-terminal. Despite the similar catalytic mechanism of the TKT family, sequence variations at the “TKT motif” residues “Thr-His-Asp” in PTKTs has been substituted with “Ser-His-Cys” in *Hs*TKT. The “TPP motif” is important in cofactor catalysis and the rearrangement of the active pocket. Short functional motif discovery predicted motif 6, 8, 12 and 16 to be uniquely conserved in *Plasmodium* sequences, but not in *Hs*TKT. Further, accurate homodimeric structures of all HI PTKTs using homology modelling were predicted. The mapping of predicted motifs on structures indicated that motif 6, 8, 12 and 16 comprise the substrate-binding pocket in PTKTs, which could indicate their functional role in substrate binding. These results formed the basis of identifying selective scaffolds against all PTKTs using molecular docking, MD simulations and BFE calculations. From the results, SANC00107, SANC00411 and SANC00620 selectively bind to all HI PTKTs and exhibited better binding affinity and molecular interactions than the known TKT inhibitor (HPP). MD simulation and BFE of identified compounds corroborate and support the molecular docking experiments. Ligand binding caused decreased RMSD profiles and conformational changes with holo–ligand bound complexes (*P. falciparum* SANC00620, *P. vivax* SANC00411 and SANC00620, *P. ovale* SANC00411 and SANC00620, *P. malariae* SANC00411 and SANC00630 and *P. knowlesi* SANC00411). As observed in VMD, the notable conformational differences were due to the highly dynamic loop regions. Using all-atom Rg, the effect of ligand binding on the active site environment revealed that the binding of ligands stabilized the active site in all systems as seen by the reduced Rg values compared to the holo proteins. Additionally, the stability of each ligand showed remained stably bound onto the protein active pocket with the exception of *P. malariae* SANC00411 complex where a flip-flop movement of the compound’s ester tail region was observed. The validation of selective ligands towards PTKTs, the dynamics of each compound in the *Hs*TKT. All three compounds exited the binding pocket at different time points. This observed event further strengthens the possibility of the identified compounds showing selective inhibition against the PTKTs. BFE favoured binding of identified compounds to proteins. This study additionally proposes a suitable solute dielectric constant (pdie) of 4 for calculating polar solvation energy of PTKTs due to the charged nature of the active sites. These hits compounds are promising, presenting a range of potential basics to novel PTKT inhibitors scaffolds, which can be used to design better PTKTs inhibitors.

## Supplementary information

**Additional file 1.** A summary of TKT homologs isolated from *Plasmodium* species and the *Homo sapiens*.

**Additional file 2.** A summarized table of plasmodial, *Homo sapiens* and other TKT sequences retrieved and their reverse BLAST results. *Indicates the query sequence and R indicate the reverse Blast results.

**Additional file 3.** Template selection for model of *Pf*TKT, *Pv*TKT, *Po*TKT, *Pm*TKT and *Pk*TKT. The best template selected is indicated in *.

**Additional file 4.** Motif locations unique to protozoans including plasmodial TKTs. -Indicates motif not found in *Hs*TKT.

**Additional file 5.** Interactions between plasmodial TKTs and SANC00107 hit compound. Hydrogen interactions are indicated in green dash lines.

**Additional file 6.** Interactions between plasmodial TKTs and SANC00411 compound. Hydrogen interactions are indicated in green dash lines.

**Additional file 7.** Interactions between plasmodial TKTs and SANC00620 compound. Hydrogen interactions are indicated in green dash lines.

**Additional file 8.** Line gragh of backbone RMSD values. Both holo an holo–ligand bound systems are represented. In yellow are each ligands in each system.

**Additional file 9.** Density distribution plots of backbone Cα RMSD values. Both hit-free and hit-bound systems are represented. Several conformations can be identified by comparing the the Cα RMSD distribution of the holo and holo–ligand bound systems.

**Additional file 10.** Free energy landscapes of conformational distribution of each system snapshot determined using the Boltzmann constant and plotted along the RMSD and Rg. In the systems showing more than one stable conformation (lowest energy), structure snapshot were generated in each energy minima and visualized using PyMOL.

**Additional file 11.** Statistics distribution of RMSD values. The means of each ligand bound complex was compared to the ligand-free system using the z-test statistic with α = 0.05 and a null hypothesis of H1–H2 = 0. The hartigan’s dip test statistic for unimodality was computed at conf.level of 0.50 and the null hypothesis (RMSD distribution unimodal distribution).

**Additional file 12.** Time dependent hydrogen bonds formed with *Pv*TKT. Hydrogen bonds shown as blue lines were calculated at 25 ns, 50 ns, 75 ns and 100 ns snapshots using Plip tool. A; SANC00107, B; SANC00411 and C; SANC00620.

**Additional file 13.** Time dependent hydrogen bonds formed with *Po*TKT. Hydrogen bonds shown as blue lines were calculated at 25 ns, 50 ns, 75 ns and 100 ns snapshots using Plip tool. A; SANC00107, B; SANC00411 and C; SANC00620.

**Additional file 14.** Time dependent hydrogen bonds formed with *Pm*TKT. Hydrogen bonds shown as blue lines were calculated at 25 ns, 50 ns, 75 ns and 100 ns snapshots using Plip tool. A; SANC00107, B; SANC00411 and C; SANC00620.

**Additional file 15.** Intermolecular interactions of each ligand–protein complex.

## Data Availability

All data generated or analysed during this study are included in this published article. All protein models are available from the corresponding author on reasonable request.

## Abbreviations

ACPYPE: AnteChamber PYthon Parser interface
BFE: Binding free energy
GROMACS: GROningen MAchine for Chemical Simulations
HI: Human infected
HPP: *p*-Hydroxyphenylpyruvate
MD: Molecular dynamics
MM-PBSA: Molecular mechanics-Poisson–Boltzmann surface area
MSA: Multiple sequence alignment
NCBI: National Center for Biotechnology Information
PlasmoDB: Plasmodium genome database
PTKTs: Plasmodial transketolases
RI: Rodent infected
SANCDB: South African natural compound database
TKT: Transketolase
