# Supplementary material for: Characterisation of plasmodial transketolases and identification of potential inhibitors: an in silico study
Source: Malar J. 2020 Nov 30;19:442. doi: 10.1186/s12936-020-03512-1 (PMC7756947; doi:10.1186/s12936-020-03512-1)
Supplement: Supplementary file 1 — Additional file 1. A summary of TKT homologs isolated from Plasmodium species and the Homo sapiens. [file 12936_2020_3512_MOESM1_ESM.docx]

**Additional file 1.** A summary of TKT homologs isolated from *Plasmodium* species and the *Homo sapiens.*

| Proteins of interest | | | | |
| --- | --- | --- | --- | --- |
| Source organism | Sequence ID | Abbreviated name | Sequence identity (%) | Expected value |
| *Plasmodium falciparum* 3D7 | PF3D7_0610800.1 | *Pf*TKT | 100 | 0.00 |
| *Plasmodium vivax* | PVPO1_1138400.1 | *Pv*TKT | 79 | 0.00 |
| *Plasmodium ovale* | ProGH01_11046100.1 | *Po*TKT | 81 | 0.00 |
| *Plasmodium knowlesi* strain H | PKNH_11396100.1 | *Pk*TKT | 80 | 0.00 |
| *Plasmodium malariae* | PmUG01_11052300.1 | *Pm*TKT | 84 | 0.00 |
| *Plasmodium yoelii* strain 17XNL | PY03111_t26_1 | *Py*TKT | 78 | 0.00 |
| *Plasmodium berghei* strain ANKA | PBANKA_0109100.1 | *Pb*TKT | 78 | 0.00 |
| *Plasmodium chabaudi chabaudi* | PCHAS_0109700.1 | *Pc*TKT | 77 | 0.00 |
| *Homo sapiens* | NP_001244957.1 | *Hs*TKT | 28 | 1e-44 |

**Additionally, retrieved sequences**

| **TKT proteins** | **Sequence ID** | **Abbreviated name** | **Sequence identity (%)** | **Expected value** |
| --- | --- | --- | --- | --- |
| ***Trypanosoma cruzi*** | EKG07569.1 | *Tc*TKT | 45 | 0.00 |
| ***Trypanosoma vivax*** | CC49969.1 | *Tv*TKT | 44 | 0.00 |
| ***Leishmania mexicana*** | XP_003876024.1 | *Lm*TKT | 43 | 0.00 |
| ***Leishmania donovani*** | XP_003861304.1 | *Ld*TKT | 43 | 0.00 |
| ***Neisseria gonorrhoea*** | WP_003688227.1 | *Ng*TKT | 41 | 3e-163 |
| ***Escherichia coli*** | WP_0878988261.1 | *Ec*TKT | 41 | 3e-162 |
| ***Chlamydomonas reinhardtii*** | XP_001701881.1 | *Cr*TKT | 43 | 0.00 |
| ***Saccharomyces cerevisiae*** | GAX68916 | *Sc*TKT | 49 | 0.00 |
| ***Anopheles darlingi*** | ETN59360.1 | *Ad*TKT | 26 | 5e-31 |
