# Supplementary material for: Characterisation of plasmodial transketolases and identification of potential inhibitors: an in silico study
Source: Malar J. 2020 Nov 30;19:442. doi: 10.1186/s12936-020-03512-1 (PMC7756947; doi:10.1186/s12936-020-03512-1)
Supplement: Supplementary file 2 — Additional file 2. A summarized table of plasmodial, Homo sapiens and other TKT sequences retrieved and their reverse BLAST results. *Indicates the query sequence and R indicate the reverse Blast results. [file 12936_2020_3512_MOESM2_ESM.docx]

**Additional file 2.** A summarized table of plasmodial, *Homo sapiens* and other TKT sequences retrieved and their reverse BLAST results. ⃰ Indicates the query sequence and R indicate the reverse Blast results.

| **Protein name (annotated)** | **Abbreviated names** | **Accession Number** | **BLAST** | **Total score** | **E-value** | **Sequence identity (%)** |
| --- | --- | --- | --- | --- | --- | --- |
| ***Plasmodium falciparum⃰*** | *Pf*TKT | PF3D7_0610800 | R | 1401  1401 | 0.0  0.0 | 100  100 |
| ***Plasmodium vivax*** | *Pv*TKT | PVP01_1138400.1 | R | 1129  1400 | 0.0  0.0 | 79  100 |
| ***Plasmodium ovale*** | *Po*TKT | PocGH01_11046100.1 | R | 1149  1408 | 0.0  0.0 | 81  100 |
| ***Plasmodium malariae*** | *Pm*TKT | PmUG01_11052300.1 | R | 1202  1402 | 0.0  0.0 | 84  100 |
| ***Plasmodium knowlesi*** | *Pk*TKT | PKNH_1139600.1 | R | 1150  1402 | 0.0  0.0 | 80  100 |
| ***Plasmodium berghei*** | *Pb*TKT | PBANKA_0109100.1 | R | 1121  1388 | 0.0  0.0 | 78  100 |
| ***Plasmodium chabaudi*** | *Pc*TKT | PCHAS_0109700.1 | R | 1104  1389 | 0.0  0.0 | 77  100 |
| ***Plasmodium yoelii*** | *Py*TKT | PY03111-t26_1-p1 | R | 1123  1390 | 0.0  0.0 | 78  100 |
| ***Homo sapiens*** | *Hs*TKT | NP_001244957.1 | R | 174  1313 | 1e-45  0.0 | 28  100 |
| ***Leishmania donovani*** | *Ld*TKT | XP_003861304.1 | R | 564  1393 | 0.0  0.0 | 43  100 |
| ***Leishmania mexicana*** | *Lm*TKT | XP_003876024.1 | R | 1391 | 0.0  0.0 | 43  100 |
| ***Trypanosoma cruzi*** | *Tc*TKT | EKG07569.1 | R | 567  1399 | 0.0  0.0 | 44  100 |
| ***Trypanosoma vivax*** | *Tv*TKT | CCC49969.1 | R | 583  1383 | 0.0  0.0 | 45  100 |
| ***Saccharomyces cerevisiae*** | *Sc*TKT | GAX68916 | R | 636  1400 | 0.0  0.0 | 49  100 |
| ***Anopheles darlingi*** | *Ad*TKT | ETN59360.1 | R | 129  1300 | 5e-31  0.0 | 26  100 |
| ***Neisseria gonorrhoea*** | *Ng*TKT | WP_003688227.1 | R | 480  1370 | 7e-162  0.0 | 41  100 |
| ***Escherichia coli*** | *Ec*TKT | WP_087898826.1 | R | 486  1385 | 4e-162  0.0 | 41  100 |
| ***Chlamydomonas reinhardtii*** | *Cr*TKT | XP_001701881.1 | R | 558  1495 | 0.0  0.0 | 43  100 |
