# Supplementary material for: Characterisation of plasmodial transketolases and identification of potential inhibitors: an in silico study
Source: Malar J. 2020 Nov 30;19:442. doi: 10.1186/s12936-020-03512-1 (PMC7756947; doi:10.1186/s12936-020-03512-1)
Supplement: Supplementary file 3 — Additional file 3. Template selection for model of PfTKT, PvTKT, PoTKT, PmTKT and PkTKT. The best template selected is indicated in *. [file 12936_2020_3512_MOESM3_ESM.docx]

**Additional file 3.** Template selection for model of *Pf*TKT, *Pv*TKT, *Po*TKT, *Pm*TKT and *Pk*TKT. The best template selected is indicated in ⃰.

| **PDB ID** | **Resolution**  **(Å)** | **Coverage**  **(%)** | **Sequence identity**  **(%)** | **Missing residues in PDB** |
| --- | --- | --- | --- | --- |
| **1TRK⃰** | 2.00 | 6-668 (98) | 49 | 2 |
| **1TKA** | 2.70 | 6-668 (98) | 49 | 13 |
| **1AY0** | 2.60 | 6-668 (98) | 49 | 2 |
| **1ITZ** | 2.30 | 6-666 (98) | 45 | 9 |
| **1R9J** | 2.22 | 11-661 (96) | 43 | 3 |
| **2E6K** | 2.09 | 6-667 (98) | 42 | 4 |
| **4C7V** | 2.20 | 6-664 (98) | 42 | 19 |
| **3HYL** | 2.17 | 2-663 (98) | 43 | 25 |
| **4XEU** | 1.95 | 14-666 (96) | 40 | 30 |
