# Supplementary material for: Characterisation of plasmodial transketolases and identification of potential inhibitors: an in silico study
Source: Malar J. 2020 Nov 30;19:442. doi: 10.1186/s12936-020-03512-1 (PMC7756947; doi:10.1186/s12936-020-03512-1)
Supplement: Supplementary file 4 — Additional file 4. Motif locations unique to protozoans including plasmodial TKTs. -Indicates motif not found in HsTKT. [file 12936_2020_3512_MOESM4_ESM.docx]

**Additional file 4.** Motif locations unique to protozoans including plasmodial TKTs. -Indicates motif not found in *Hs*TKT

| **Motifs (M)** | ***Pf*TKT** | ***Pv*TKT** | ***Po*TKT** | ***Pm*TKT** | ***Pk*TKT** | ***HsTKT*** |
| --- | --- | --- | --- | --- | --- | --- |
| **M6** | 406-425 | 403-422 | 403-422 | 406-425 | 403-422 | - |
| **M8** | 186-205 | 183-202 | 183-202 | 186-205 | 183-202 | - |
| **M10** | 586-605 | 581-600 | 482-531 | 586-605 | 482-531 | - |
| **M12** | 374-393 | 377-390 | 377-390 | 374-393 | 377-390 | - |
| **M13** | 636-655 | 634-653 | 634-653 | 636-655 | 634-653 | - |
| **M16** | 515-534 | 512-531 | 512-531 | 515-534 | 512-531 | - |
| **M18** | 563-572 | 551-570 | 551-570 | 563-572 | 551-570 | - |
| **M19** | 326-345 | 323-342 | 323-342 | 326-345 | 323-342 | - |
| **M20** | 614-633 | 612-631 | 612-631 | 614-633 | 612-631 | - |
| **M21**  **M24**  **M25** | 7-26  174-184  133-148 | 4-23  170-181  130-145 | 4-23  170-181  130-145 | 7-26  174-184  133-148 | 4-23  170-181  130-145 | - |

**Length of motifs unique to plasmodial TKTs**

| **M26** | 306-325 | 303-322 | 303-322 | 306-325 | 303-322 | - |
| --- | --- | --- | --- | --- | --- | --- |
| **M27** | 535-549 | 532-546 | 532-546 | 535-549 | 532-546 | - |
| **M30** | 394-405 | 391-402 | 391-402 | 394-405 | 391-402 | - |
|  |  |  |  |  |  |  |
