# Supplementary material for: Characterisation of plasmodial transketolases and identification of potential inhibitors: an in silico study
Source: Malar J. 2020 Nov 30;19:442. doi: 10.1186/s12936-020-03512-1 (PMC7756947; doi:10.1186/s12936-020-03512-1)

**Additional file 6.** Interactions between plasmodial TKTs and SANC00411 compound. Hydrogen interactions are indicated in green dash lines.


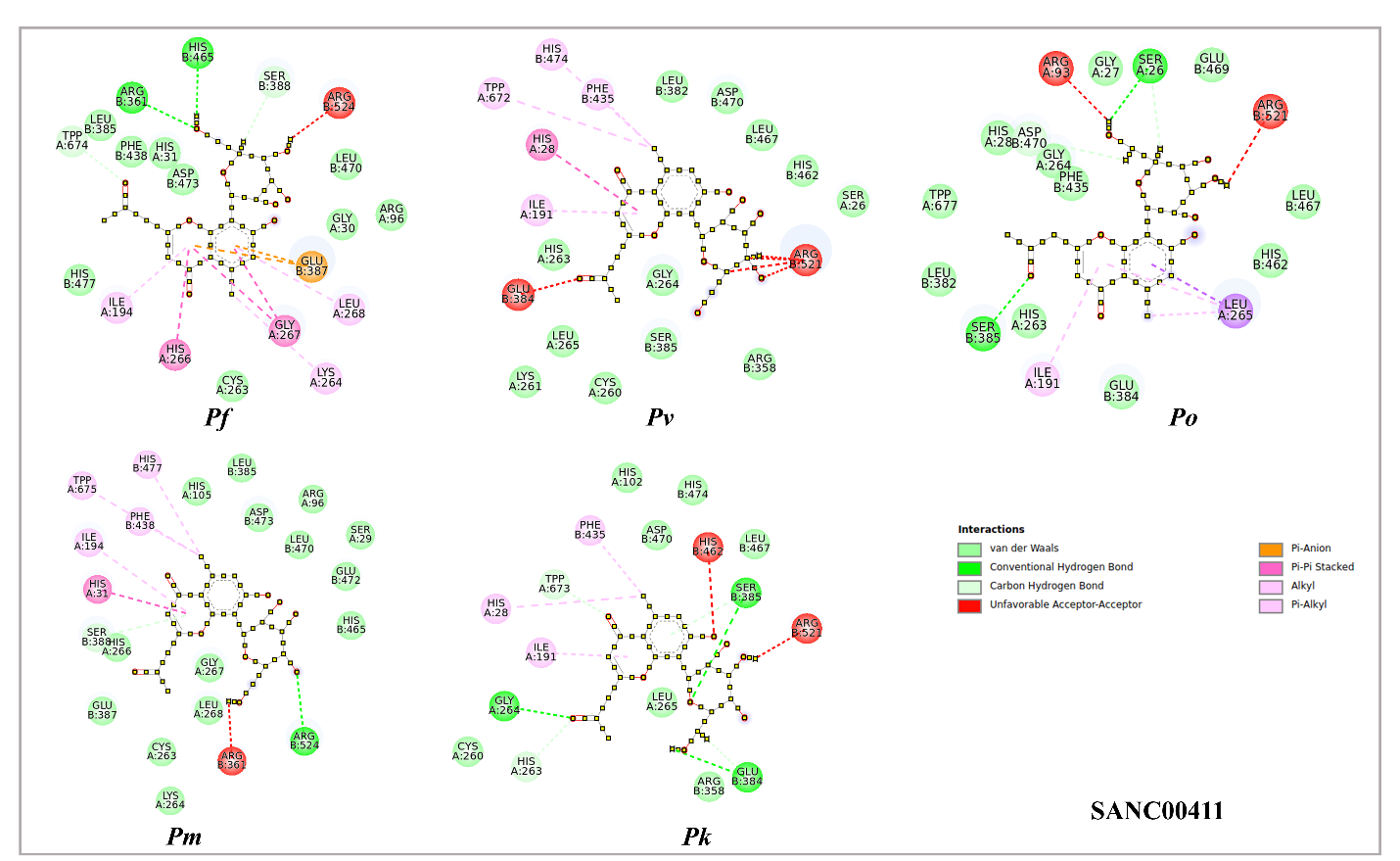

Supplement: Supplementary file 6 — Additional file 6. Interactions between plasmodial TKTs and SANC00411 compound. Hydrogen interactions are indicated in green dash lines. [file 12936_2020_3512_MOESM6_ESM.docx]
