# Supplementary material for: Characterisation of plasmodial transketolases and identification of potential inhibitors: an in silico study
Source: Malar J. 2020 Nov 30;19:442. doi: 10.1186/s12936-020-03512-1 (PMC7756947; doi:10.1186/s12936-020-03512-1)

**Additional file 8.** Line gragh of backbone RMSD values. Both holo an holo-ligand bound systems are represented. In yellow are each ligands in each system.


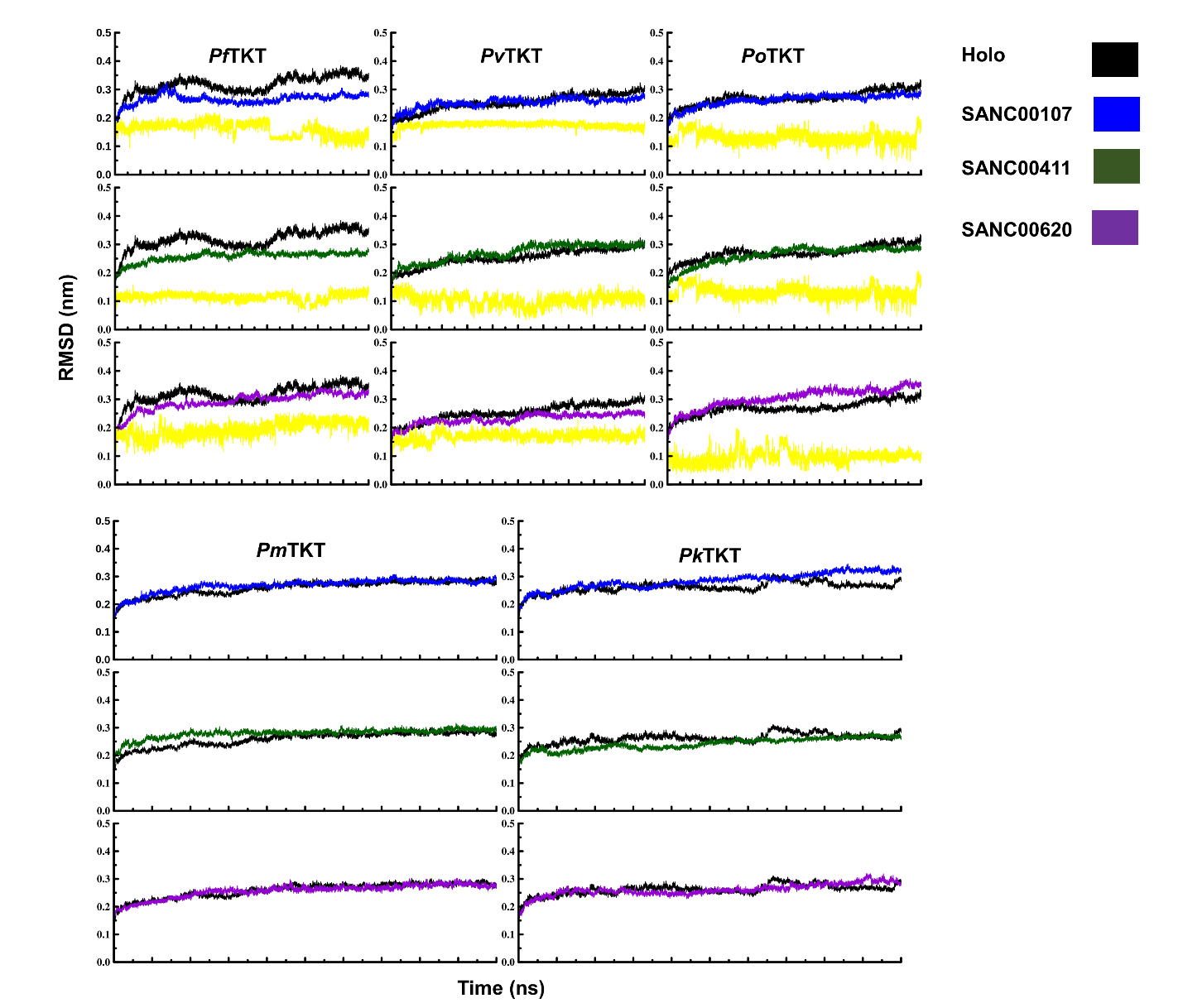

Supplement: Supplementary file 8 — Additional file 8. Line gragh of backbone RMSD values. Both holo an holo–ligand bound systems are represented. In yellow are each ligands in each system. [file 12936_2020_3512_MOESM8_ESM.docx]
