# Supplementary material for: Characterisation of plasmodial transketolases and identification of potential inhibitors: an in silico study
Source: Malar J. 2020 Nov 30;19:442. doi: 10.1186/s12936-020-03512-1 (PMC7756947; doi:10.1186/s12936-020-03512-1)

**Additional file 9.** Density distribution plots of backbone Cα RMSD values. Both hit-free and hit-bound systems are represented. Several conformations can be identified by comparing the the Cα RMSD distribution of the holo and holo-ligand bound systems.


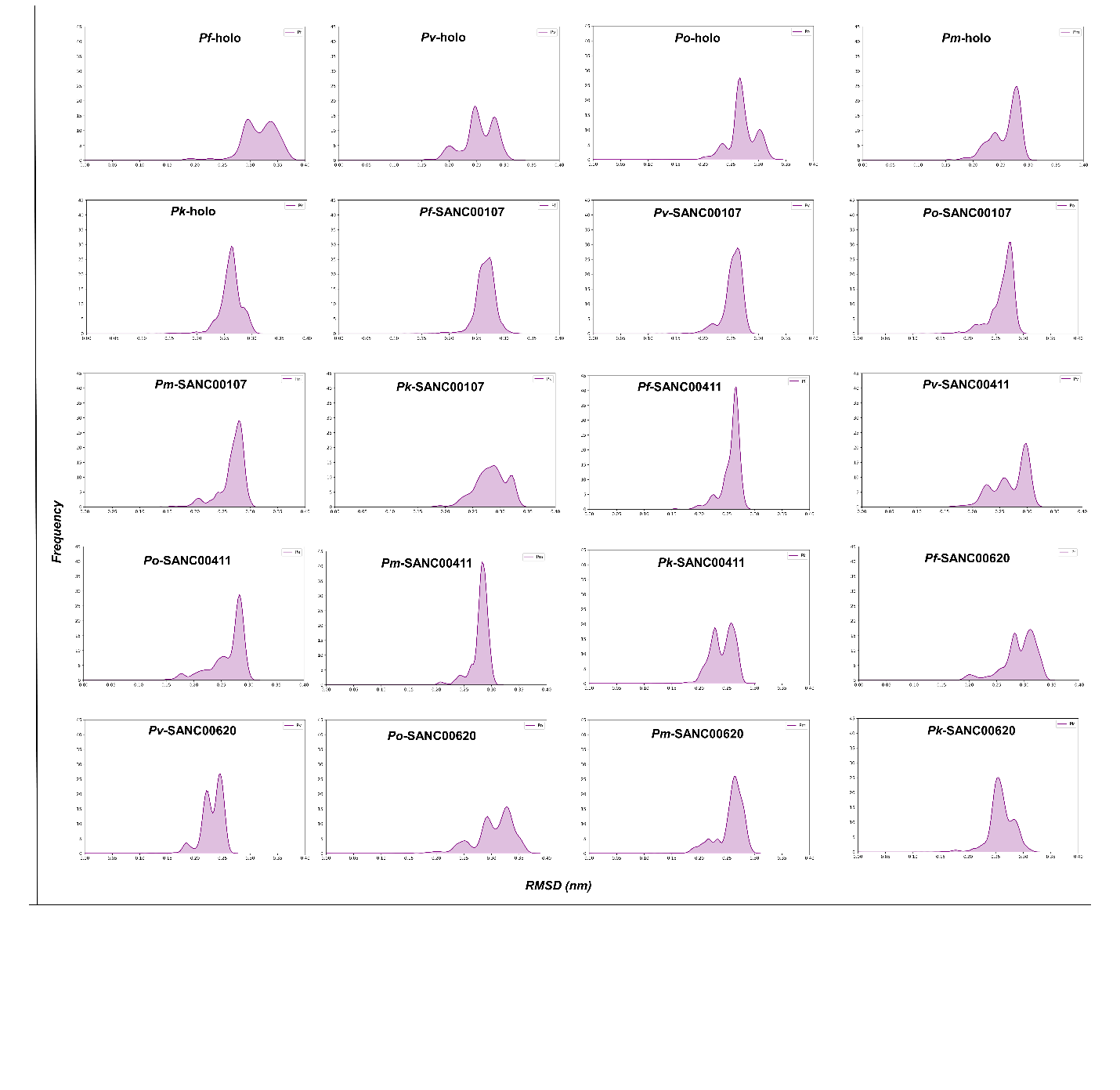

Supplement: Supplementary file 9 — Additional file 9. Density distribution plots of backbone Cα RMSD values. Both hit-free and hit-bound systems are represented. Several conformations can be identified by comparing the the Cα RMSD distribution of the holo and holo–ligand bound systems. [file 12936_2020_3512_MOESM9_ESM.docx]
