# Supplementary material for: Characterisation of plasmodial transketolases and identification of potential inhibitors: an in silico study
Source: Malar J. 2020 Nov 30;19:442. doi: 10.1186/s12936-020-03512-1 (PMC7756947; doi:10.1186/s12936-020-03512-1)

**Additional file 10.** Free energy landscapes of conformational distribution of each system snapshot determined using the Boltzmann constant and plotted along the RMSD and Rg. In the systems showing more than one stable conformation (lowest energy), structure snapshot were generated in each energy minima and visualized using PyMOL.


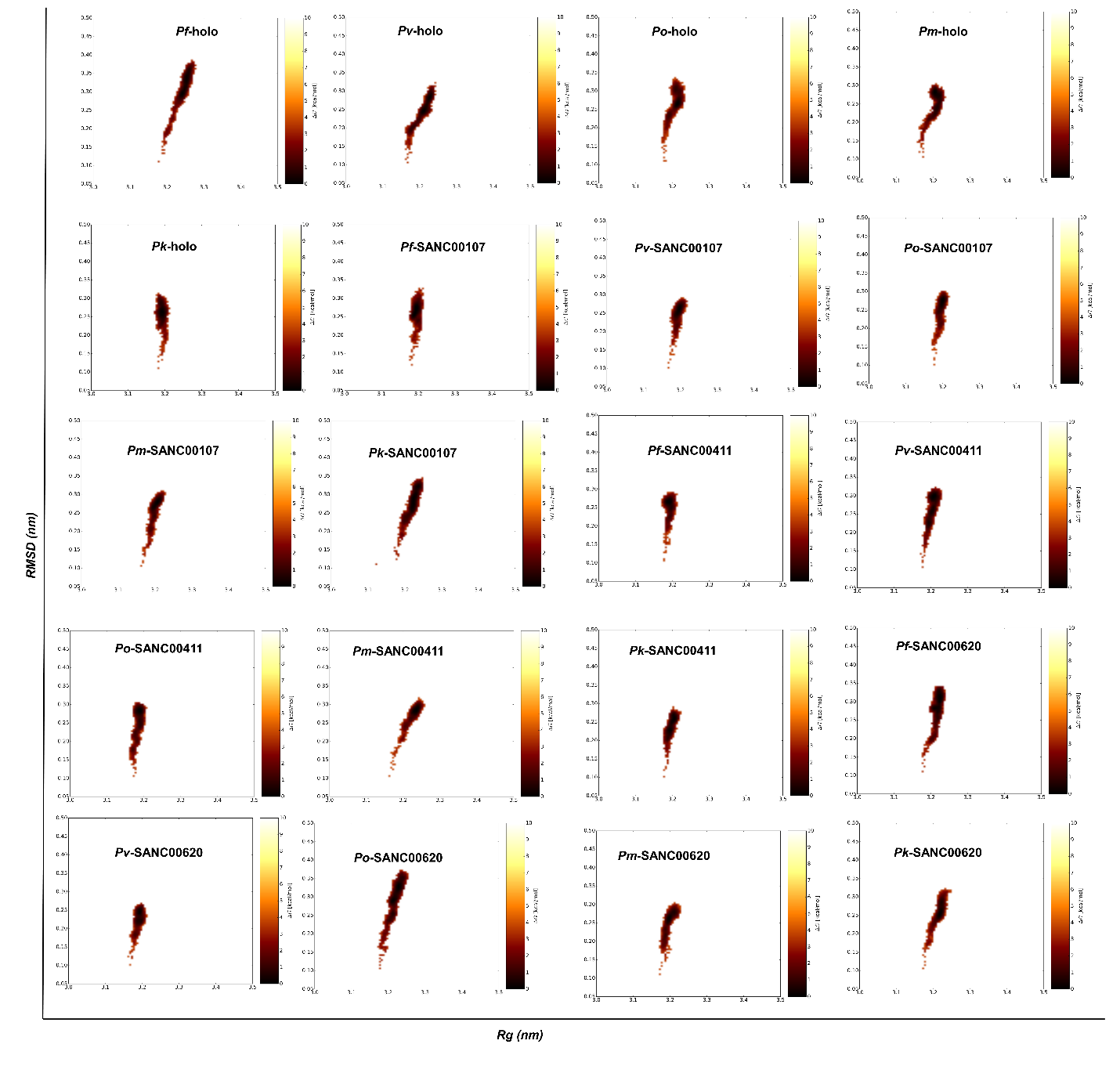

Supplement: Supplementary file 10 — Additional file 10. Free energy landscapes of conformational distribution of each system snapshot determined using the Boltzmann constant and plotted along the RMSD and Rg. In the systems showing more than one stable conformation (lowest energy), structure snapshot were generated in each energy minima and visualized using PyMOL. [file 12936_2020_3512_MOESM10_ESM.docx]
