# Supplementary material for: Characterisation of plasmodial transketolases and identification of potential inhibitors: an in silico study
Source: Malar J. 2020 Nov 30;19:442. doi: 10.1186/s12936-020-03512-1 (PMC7756947; doi:10.1186/s12936-020-03512-1)
Supplement: Supplementary file 11 — Additional file 11. Statistics distribution of RMSD values. The means of each ligand bound complex was compared to the ligand-free system using the z-test statistic with α = 0.05 and a null hypothesis of H1–H2 = 0. The hartigan’s dip test statistic for unimodality was computed at conf.level of 0.50 and the null hypothesis (RMSD distribution unimodal distribution). [file 12936_2020_3512_MOESM11_ESM.docx]

**Additional file 11.** Statistics distribution of RMSD values. The means of each ligand bound complex was compared to the ligand-free system using the z-test statistic with α = 0.05 and a null hypothesis of H1 – H2 = 0. The hartigan's dip test statistic for unimodality was computed at conf.level of 0.50 and the null hypothesis (RMSD distribution unimodal distribution).

| Protein | Compounds | Mode | Var. | Z-test | | Hartigans dip test for distribution |
| --- | --- | --- | --- | --- | --- | --- |
|  |  |  |  |  | p-value | p-value |
|  | Holo-protein | 0.32 | 0.00 | - | - | 2.2e^-16^ |
| *PfT*KT | SANC00107 | 0.27 | 0.00 | 138.9 | 0.00 | 0.99 |
|  | SANC00411 | 0.26 | 0.00 | 61.11 | 0.00 | 0.83 |
|  | SANC00620 | 0.29 | 0.00 | 56.95 | 0.00 |  |
|  |  |  |  |  |  |  |
| *Pv*TKT | Holo-protein | 0.26 | 0.00 | - | - | 2.2e^-16^ |
|  | SANC00107 | 0.25 | 0.00 | 1.82 | 0.07 | 0.86 |
|  | SANC00411 | 0.27 | 0.00 | -36.81 | 0.00 | 2.2e^-16^ |
|  | SANC00620 | 0.23 | 0.00 | 64.12 | 0.00 | 2.2e^-16^ |
|  |  |  |  |  |  |  |
| *Po*TKT | Holo-protein | 0.27 | 0.00 | - | - | 2.2e^-16^ |
|  | SANC00107 | 0.26 | 0.00 | 23.77 | 0.00 | 0.9955 |
|  | SANC00411 | 0.26 | 0.00 | 20.2 | 0.00 | 0.22 |
|  | SANC00620 | 0.30 | 0.00 | -74.76 | 0.00 | 2.2e^-16^ |
|  |  |  |  |  |  |  |
| *Pm*TKT | Holo-protein | 0.26 | 0.00 | - | - | 2.2e^-16^ |
|  | SANC00107 | 0.27 | 0.00 | -21.65 | 0.00 | 0.18 |
|  | SANC00411 | 0.28 | 0.00 | -62.10 | 0.00 | 0.90 |
|  | SANC00620 | 0.25 | 0.00 | 7.64 | 0.00 | 0.67 |
|  |  |  |  |  |  |  |
| *Pk*TKT | Holo-protein | 0.26 | 0.00 | - | - | 0.10 |
|  | SANC00107 | 0.28 | 0.00 | -61.05 | 0.00 | 2.2e^-16^ |
|  | SANC00411 | 0.25 | 0.00 | 70.76 | 0.00 | 2.2e^-16^ |
|  | SANC00620 | 0.23 | 0.00 | 3.96 | 0.00 | 0.403 |
