# Supplementary material for: Characterisation of plasmodial transketolases and identification of potential inhibitors: an in silico study
Source: Malar J. 2020 Nov 30;19:442. doi: 10.1186/s12936-020-03512-1 (PMC7756947; doi:10.1186/s12936-020-03512-1)

**Additional file 14.** Time dependent hydrogen bonds formed with *Pm*TKT. Hydrogen bonds shown as blue lines were calculated at 25ns, 50ns, 75ns and 100ns snapshots using Plip tool. A; SANC00107, B; SANC00411 and C; SANC00620.


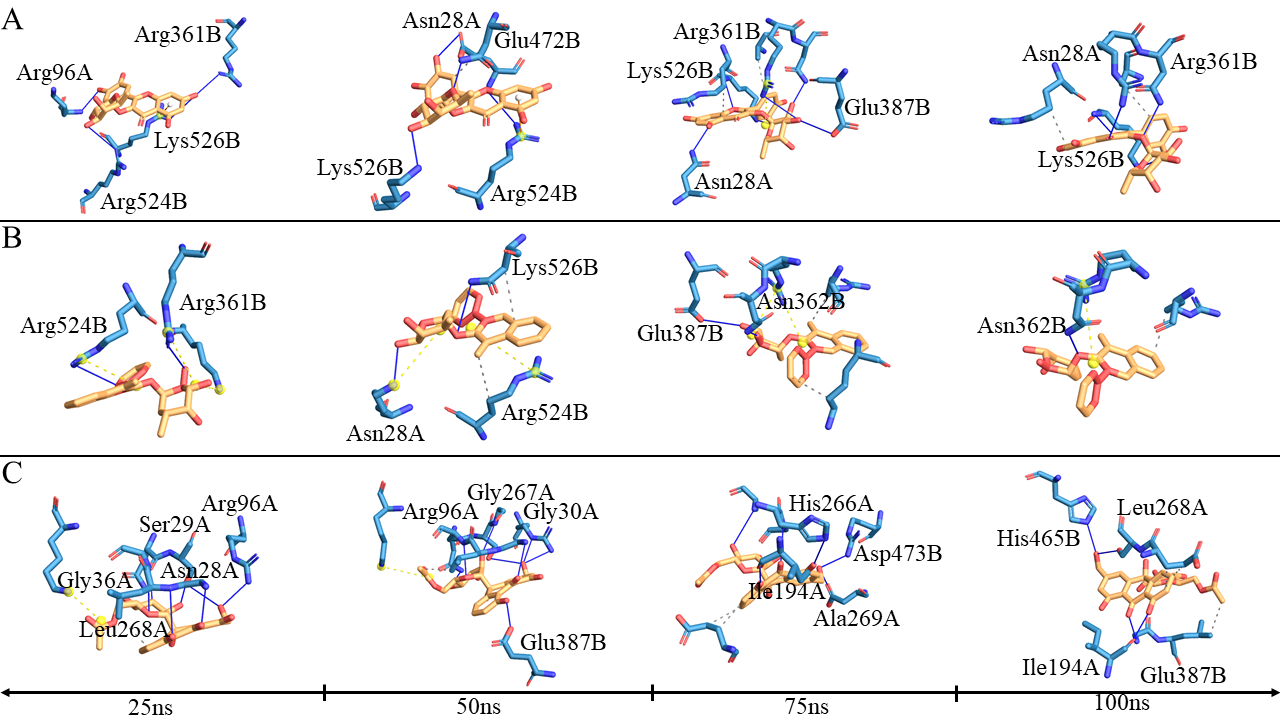

Supplement: Supplementary file 14 — Additional file 14. Time dependent hydrogen bonds formed with PmTKT. Hydrogen bonds shown as blue lines were calculated at 25 ns, 50 ns, 75 ns and 100 ns snapshots using Plip tool. A; SANC00107, B; SANC00411 and C; SANC00620. [file 12936_2020_3512_MOESM14_ESM.docx]
