# Supplementary material for: Characterisation of plasmodial transketolases and identification of potential inhibitors: an in silico study
Source: Malar J. 2020 Nov 30;19:442. doi: 10.1186/s12936-020-03512-1 (PMC7756947; doi:10.1186/s12936-020-03512-1)
Supplement: Supplementary file 15 — Additional file 15. Intermolecular interactions of each ligand–protein complex. [file 12936_2020_3512_MOESM15_ESM.docx]

**Additional file 15****.** Intermolecular interactions of each ligand-protein complex.

| Protein | Compounds |  | Hydrophobic Interactions | Pi-stacking | Pi-cation | Salt bridges |
| --- | --- | --- | --- | --- | --- | --- |
| *PfT*KT | SANC00107 |  | Ile194, Asp 195, Leu385 and Glu387 |  |  |  |
|  | SANC00411 |  | Arg 542 |  | Arg361 and Arg524 |  |
|  | SANC00620 |  | Glu387, Phe438 and Leu470 |  |  | Arg96 |
| *Pv*TKT | SANC00107 |  | Glu384 and Phe435 |  | His263 |  |
|  | SANC00411 |  | Ile191 and Glu384 | His28 | His28 |  |
|  | SANC00620 |  | Leu 264, Leu382 and Glu384 |  | His 28 |  |
| *Po*TKT |  |  |  |  |  |  |
|  | SANC00107 |  | Glu384, Phe435 |  | His 263 |  |
|  | SANC00411 |  | Ile191, His263, Leu265, His263, Leu265, Leu382 |  |  |  |
|  | SANC00620 |  | Ile191, Leu382, Leu467 |  |  | His28 |
| *Pm*TKT |  |  |  |  |  |  |
|  | SANC00107 |  | Asn28, Ala359 and Arg524 |  | Arg524 | Arg361 and Arg 524 |
|  | SANC00411 |  | Asn28, Arg524 and Lys526 |  |  | Arg361, Arg524 and Lys526 |
|  | SANC00620 |  | Glu387 and Leu268 |  |  | Lys271 |
| *Pk*TKT |  |  |  |  |  |  |
|  | SANC00107 |  | Ile191, Leu265, Leu382, Glu284 and Phe435 |  |  | Ly271 |
|  | SANC00411 |  | Leu22, His28, Ile191, Leu265, Leu267, Leu382, Glu384 |  |  |  |
|  | SANC00620 |  | Ile191 and Thr461 |  |  | His462 and Arg521 |
